# Exploring the relationship between maternal carbohydrate quality and quantity during pregnancy and early childhood neurodevelopment: a prospective cohort study within the BiSC cohort

**DOI:** 10.1007/s00394-025-03829-0

**Published:** 2025-12-01

**Authors:** Laura Panisello, Javier Mateu-Fabregat, Nil Novau-Ferré, Nicolas Ayala-Aldana, Sara Bernardo-Castro, Muriel Ferrer, Pol Jiménez-Arenas, Elisa Llurba, Camille Lassale, María Dolores Gómez-Roig, Jesús Vioque, Sandra González-Palacios, Oren Contreras-Rodríguez, Maria Foraster, Mireia Gascon, Jordi Sunyer, Camila Awad, Jordi Júlvez, Mònica Bulló

**Affiliations:** 1 Nutrition and Metabolic Health Research Group, Department of Biochemistry and Biotechnology, Rovira i Virgili University (URV), 43204 Reus, Spain; 2 Institute of Health Pere Virgili (IISPV), 43204 Reus, Spain; 3 Center of Environmental, Food and Toxicological Technology—TecnATox, Rovira i Virgili University, 43204 Reus, Spain; 4 Clinical and Epidemiological Neuroscience (NeuroÈ̇pia), IISPV, Reus, Spain; 5 ISGlobal, Barcelona, Spain; 6 University of Barcelona, Barcelona, Spain; 7 Universitat Pompeu Fabra (UPF), Barcelona, Spain; 8 CIBER de Epidemiología y Salud Pública (CIBERESP), Carlos III Health Institute, Madrid, Spain; 9 Department of Obstetrics and Gynecology, Institut d’Investigació Biomèdica Sant Pau-IIB Sant Pau, Hospital de la Santa Creu i Sant Pau, Barcelona, Spain; 10 CIBER Physiology of Obesity and Nutrition (CIBEROBN), Carlos III Health Institute, Madrid, Spain; 11 BCNatal. Barcelona Center for Maternal Fetal and Neonatal Medicine (Hospital Sant Joan de Déu and Hospital Clínic), University of Barcelona, Barcelona, Spain; 12 Institut de Recerca Sant Joan de Déu, Esplugues de Llobregat, Barcelona, Spain; 13 Primary Care Interventions to Prevent Maternal and Child Chronic Diseases of Perinatal and Developmental Origin Network (RICORS), RD21/0012/0003, Instituto de Salud Carlos III, Madrid, Spain; 14 Universidad Miguel Hernández (UMH), Alicante, Spain; 15 Instituto de Investigación Sanitaria y Biomédica de Alicante (ISABIAL), Alicante, Spain; 16 Department of Psychiatry and Forensic Medicine, Universitat Autònoma de Barcelona, Bellaterra, Spain; 17 CIBER Mental Health (CIBERSAM), Carlos III Health Institute, Madrid, Spain; 18 PHAGEX Research Group, Blanquerna School of Health Science, Universitat Ramon Llull (URL), Barcelona, Spain; 19 Unitat de Suport a La Recerca de La Catalunya Central, Fundació Institut Universitari per a la Recerca a l’Atenció Primària de Salut Jordi Gol i Gurina (IDIAPJGol), Manresa, Spain

**Keywords:** Maternal nutrition, Neurodevelopment, Carbohydrate intake, Glycemic index, Early childhood

## Abstract

**Purpose:**

Maternal nutrition during pregnancy is key for offspring neurodevelopment. Given the role of glucose in brain function, assessing carbohydrate quantity and quality, including glycemic index (GI), glycemic load (GL) and carbohydrate quality index (CQI), may provide insights into early brain development. This study examined the associations between maternal dietary carbohydrate intake and neurodevelopmental outcomes in early childhood.

**Methods:**

The prospective cohort study included 1080 mother–child pairs from the Barcelona Life Study Cohort. Maternal dietary carbohydrate intake, GI, GL and CQI were assessed during mid-pregnancy using a food frequency questionnaire. Child neurodevelopment was evaluated at 8 and 28 months using the Developmental Profile 3 (DP-3) and at 18 months using the Bayley Scales of Infant and Toddler Development (BSID-III). Associations were analyzed using multivariable linear regression models adjusted for relevant maternal and child covariates.

**Results:**

Increased maternal carbohydrate intake, GI, and GL were inversely associated with language development (β (95% CI): − 2.67 (− 5.13, − 0.21), − 2.73 (− 5.21, − 0.26), − 3.51 (− 5.96, − 1.07) respectively) and receptive language (β (95% CI): − 0.58 (− 1.07, − 0.08), − 0.54 (− 1.04, − 0.04), − 0.70 (− 1.20, − 0.21) respectively) at 18 months, as measured by the BSID-III, although these associations were attenuated after adjustment for maternal and child covariates. Increased GI and lower CQI were associated with lower gross motor scores (β (95% CI): − 0.49 (− 0.84, − 0.15), 0.39 (0.06, 0.71) respectively) at 18 months (BSID-III), as well as reduced motor development (β (95% CI): − 3.2 (− 5.50, − 0.76), 2.22 (− 0.1, 4.54) respectively) at 8 and 28 months (DP-3).

**Conclusions:**

Maternal carbohydrate quality during pregnancy may influence early neurodevelopment, particularly motor outcomes. Emphasizing low-GI, low-GL and high-CQI carbohydrate sources during pregnancy could support favorable developmental trajectories in offspring.

**Supplementary Information:**

The online version contains supplementary material available at 10.1007/s00394-025-03829-0.

## Introduction

The prenatal environment exerts a pivotal influence in shaping postnatal cognitive performance, particularly during critical windows of brain development, characterized by accelerated brain growth and heightened neural plasticity. The in-utero period is fundamental for neurogenesis and synaptogenesis. Consequently, the brain is particularly vulnerable to environmental influences, including maternal nutrition, which can exert long-term effects on cognitive outcomes [1, 2].

Extensive literature has documented the importance of several key nutrients, such as folate, iron, zinc, iodine and choline, which deficiencies during pregnancy are associated with significant offspring cognitive impairments [3, 4]. In addition to developmental disorders resulting from specific nutrient deficiencies during pregnancy, recent studies have suggested that maternal dietary patterns significantly influence offspring developmental abilities. Diets rich in docosahexaenoic acid, arachidonic acid and polyunsaturated fatty acids have been associated with improved neuromotor and language development, while higher trans fatty acid intake is associated with lower social-emotional and language scores [5]. Moreover, diets characterised by high protein and micronutrient density during pregnancy have been associated with enhanced gross motor skills and problem-solving abilities [6]. Furthermore, high adherence to the Mediterranean Diet is associated with higher cognitive and language scores, as well as a reduced likelihood of communication delays in infants [7].

Despite extensive evidence linking various macro and micronutrients to neurodevelopment, few studies have examined the relation with [8, 9] dietary carbohydrates, even though they contribute to more than half of the total energy intake in a balanced diet. Carbohydrates are the primary energy source for pregnancy, supporting fetal growth and development [10]. Glucose is essential for synthesizing compounds required for brain signalling, structural development and remodelling [11]. In adults, carbohydrates can have both beneficial and detrimental effects on cognitive health, depending on their quality. Minimally processed carbohydrates, such as whole grains, seeded bread, pulses, legumes, fruits and nuts, have been associated with a lower risk of cognitive impairment [12], while diets high in refined grains, added sugars and sweetened beverages are positively associated with an increased rate of cognitive decline [13]. Taken these findings together, we hypothesize that both the quantity and quality of maternal carbohydrate intake during pregnancy may influence early neurodevelopmental outcomes in infants. Dietary glycemic index (GI), glycemic load (GL) and carbohydrate quality index (CQI) emerge as important tools for evaluating both the amount and quality of maternal carbohydrate intake on child neurodevelopment. GI measures the rate at which glucose is released after consuming a carbohydrate containing food, relative to a reference intake such as glucose or white bread. GL complements GI by considering the available carbohydrate content per serving, providing a more comprehensive assessment of food’s impact on blood glucose level [14]. CQI evaluates carbohydrate quality by considering GI, fiber, the ratio of whole grains to total grains, and the ratio of solid carbohydrates to total carbohydrates consumed [15]. Together, GI, GL, and CQI offer a comprehensive evaluation of carbohydrate quality and its impact on glucose metabolism. Therefore, the present study aims to explore the association between the amount of maternal dietary carbohydrate intake and its quality, assessed by dietary GI, GL and CQI during pregnancy, and neurodevelopmental outcomes in children, with follow-up assessments at 8, 18 and 28 months of age.

## Methods

### Study population

The present study was conducted within the Barcelona Life Study Cohort (BiSC), which recruited 1080 mother–child pairs from the Barcelona metropolitan area between 2018 and 2021. Participants were enrolled at their first routine prenatal visit, between 11 and 15 weeks of gestation). The inclusion criteria included a singleton pregnancy, maternal age between 18 and 45 years, the ability to communicate in Spanish or Catalan, residency within the study area, and the intention to deliver at one of the three main university hospitals in Barcelona. We excluded those women residing outside the catchment area, aged < 18 years or > 45 years, illiterate, with a multiparous pregnancy or having a fetus with known congenital anomalies. The study was approved by the institutional Ethical Committees from the Parc de Salut Mar (2018/8050/I), Medical Research Committee of the Fundació de Gestió Sanitària del Hospital de la Santa Creu i Sant Pau de Barcelona (EC/18/206/5272) and Ethics Committee of the Fundació Sant Joan de Déu (PIC-27-18). All participants provided informed consent before participating [16].

**Fig. 1:**
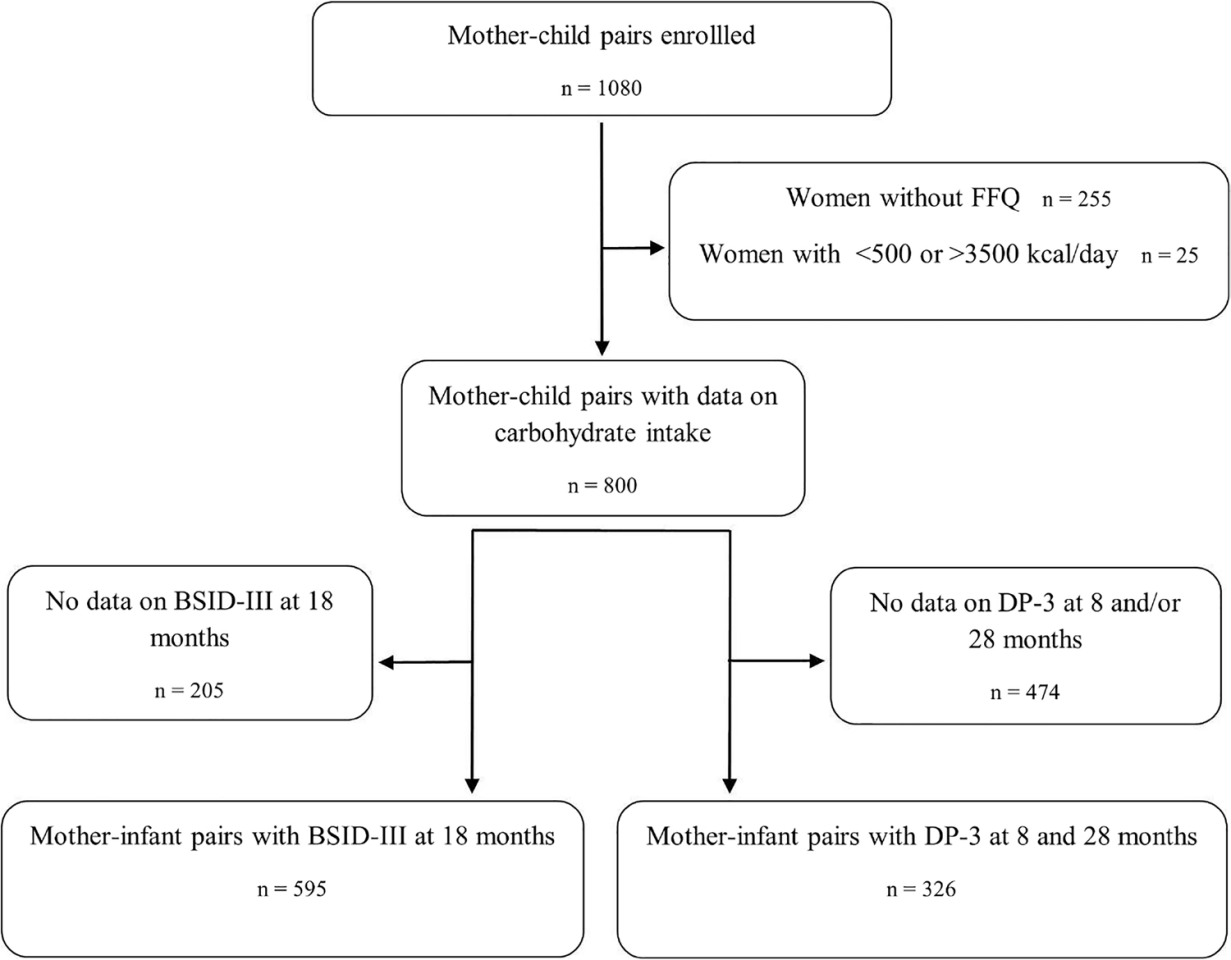
Flowchart of study population. Abbreviations: FFQ, food frequency questionnaire; BSID-III, Bayley Scales of Infant and Toddler Development; DP-3, Developmental Profile 3

The present analysis was carried out on a subset of 800 mother–child pairs, selected based on the availability of maternal dietary data assessed from completed Food Frequency Questionnaires (FFQ) and neurodevelopmental assessments of the children at different ages. Women with incompleted FFQ or implausible total energy intakes (< 500 kcal/day or > 3500 kcal/day) were excluded from the analysis [17] (Fig. 1).

### Measurements

#### Maternal carbohydrate consumption

Maternal dietary intake was assessed using a self-administered 114-item semiquantitative FFQ, specifically modified and calibrated for pregnant women and administered during the 2nd trimester of pregnancy, which collected information on dietary intake during the previous year[18]. Daily nutrient intake was estimated by multiplying the reported frequency of consumption by the standard portion size and its nutrient content, based on the Spanish food composition tables [19–21].

GI values for each FFQ item were assigned using international GI tables [22], with glucose as the reference and assigning GI values following a previously described step-based method [23]. GL was calculated by multiplying the net carbohydrate intake of each item (in grams per day) by its GI and dividing the result by 100. The total GL for each participant was the sum of the GL values across all FFQ items. Dietary GI was then determined by multiplying the total GL by 100 and dividing the result by the participant’s total carbohydrate intake (grams per day) [23]. The CQI for each participant was determined based on four components: dietary fiber intake (grams per day), dietary GI, the ratio of whole grains to total grains, and the ratio of solid carbohydrates to total carbohydrates. For each component, participants were categorized into quintiles and were given a value (score 1 to 5) according to each quintile, except GI, where high GI received lower score. Then, the overall CQI was calculated as the sum of the scores for the four components (from 4 to 20) [15].

#### Outcomes: child neurodevelopment

Child neurodevelopment was assessed using the Spanish validated version of two standardized psychometric scales: the Bayley Scales of Infant and Toddler Development, Third Edition (BSID-III) [24], and the Developmental Profile 3 (DP-3) [25]. At the 18-month follow-up hospital visit, the BSID-III was individually administered by a experienced neuropsychologist, trained in the standardized use of the Bayley scales, and blinded to maternal dietary data. This assessment evaluates three key domains of development: (i) the cognitive scale; (ii) the language scale, which includes both receptive and expressive communication; and (iii) the motor scale, measuring fine and gross motor skills [5, 26]. Raw scores for each primary scale (cognitive, language, and motor) were standardized to a mean of 100 and a standard deviation of 15, while subscales (receptive and expressive communication, and fine and gross motor skills) were standardized to a mean of 10 and a standard deviation of 3, following commonly accepted standardization practices. The DP-3 was completed by parents at the 8 and 28-month follow-ups. This DP-3 is designed to assess developmental strengths and weaknesses across five key domains: adaptive behaviour, socioemotional development, cognition, communication, and motricity. It also provides a global index of child development. Raw scores for each domain were standardised to a mean of 100 and a standard deviation of 15 [27, 28].

### Statistical analysis

The general characteristics of the study population are presented as the mean and standard deviation (SD) for normally distributed variables, while non-normally distributed variables are reported as the median and interquartile range (IQR). Categorial variables are expressed as percentages. Maternal and child characteristics across tertiles of maternal dietary GI, GL and CQI were analyzed using either the Kruskal–Wallis or chi-squared test, depending on the type of variable.

Participants were categorized into tertiles based on maternal dietary carbohydrate intake, GI, GL and CQI. The associations between these maternal dietary exposures and child neurodevelopment were evaluated using multivariate linear regression analysis. Specifically, child performance on the BSID-III at 18 month was analyzed using a classical linear model, while a linear mixed-effects model was used to assess DP-3 performance across 8- and 28-month evaluations. The p-trend for the model was calculated by incorporating the median value of each tertile in the multivariate model. In addition, the exposures were also analyzed as continuous variables to assess linear associations across the full range of values.

To examine the influence of covariates on effect estimates, results are presented across multiple models. Covariates were obtained from a combination of face-to-face interviews, online questionnaires, hospital records and clinical and physical examinations, all of them collected during pregnancy and early childhood. Missing data for covariates was handled using multiple imputations by chained equations (MICE). The quality of the imputation process was assessed by comparing observed and imputed data through density plots.

Model 1 assessed the crude association between exposure and outcome. Model 2 was adjusted for maternal age at enrolment, as well as child sex and age at the time of cognitive assessment. Model 3 included additional adjustments for a range of maternal factors; including body mass index (BMI; kg/m^2^) at enrolment, total energy intake (kcal/day) assessed at 2nd trimester of pregnancy, smoking habits during pregnancy (yes, no), socioeconomic status (medium to low and high) and education level (primary or less, secondary, university studies). Furthermore, Model 3 was also adjusted for maternal adherence to Mediterranean Diet, evaluated using the Mediterranean Diet Adherence Screener (MEDAS; categorized as a score of < 8 or ≥ 8) [29], physical activity level, assessed using the Pregnancy Physical Activity Questionnaire (PPAQ; expressed in metabolic equivalent task hours per week (MET-h/week)) [30], and the presence of gestational diabetes. Additionally, Model 3 also incorporated child-related variables, including birth weight, prematurity (yes, no), type of feeding (breastfeeding, infant formula, mixed) and delivery type (caesarean, vaginal).

Further sensitivity analysis was conducted to explore the impact of adjustment for maternal ethnicity. A secondary analysis was performed on the subset of participants with available information regarding the type of feeding at 18 months of age (breastfeeding, infant formula, mixed) (n = 565). Results from multivariate regression analysis are reported as β coefficients and their 95% confidence interval (CI). Statistical significance was set at *p*-value < 0.05. All statistical analyses were conducted using the R software (version 4.2.3; R Foundational for Statistical Computing in Vienna, Austria).

## Results

### Characteristics of study population

A total of 800 pregnant women completed the FFQ and had plausible energy intake estimates (Fig. 1). The median maternal age was 35 years old, with a BMI within the normal range (median of 23 kg/m^2^). Most women were college-educated (75.5%), non-smokers (94.7%), reported medium to low financial status (64.1%), were from Caucasian ethnicity (79.9%) and did not develop gestational diabetes (92.6%). The majority of children in the cohort were delivered vaginally (75.9%), born at term (96.5%), and had a birth weight within the normality (median of 3320 g). In the first 15 days of life, most of the children were breastfed (71.3%), while a smaller percentage were fed by infant formula (5.6%) (Table 1, S1 and S2). The median maternal carbohydrate intake was 199 g/day, contributing to 42% of the total energy intake. The median GI was 45, the GL was 91 and the CQI was 14. Among food groups, bread and cereals were the main contributors to total carbohydrate intake, dietary GI and dietary GL (Fig. S1).

**Table 1:** Characteristics of study population

*Maternal characteristics, n* = *800*	
Carbohydrate intake (g/day)	199.23 [161.09, 245.69]
Carbohydrate contribution to energy intake (%)	42.97 [38.51, 47.45]
Protein contribution to energy intake (%)	17.97 [16.15, 19.97]
Fat contribution to energy intake (%)	18.25 [16.57, 19.85]
Glycemic index (%)	44.72 [40.87, 50.06]
Glycemic load	91.25 [69.25, 118.14]
Carbohydrate Quality Index	14 [12, 16]
Age (years)	34.78 [31.85, 37.20]
Body Mass Index (kg/m^2^)	23.16 [21.18, 25.60]
Total energy intake (kcal/day)	1893.91 [1535.28, 2277.44]
Smoking during pregnancy, n (%)	
Yes	42 (5.25)
No	758 (94.75)
Financial status, n (%)	
High	287 (35.87)
Medium to low	513 (64.13)
Education level, n (%)	
Secondary school or below	196 (24.50)
University studies	604 (75.50)
Ethinicity, n (%)	
Caucasian	639 (79.88)
Other	161 (20.12)
Mediterranean Diet Adherence Screener, n (%)	
High adherence (≥ 8)	478 (59.75)
Low adherence (< 8)	322 (40.25)
Pregnancy Physical Activity Questionnaire (MET-h/week)	163.11 [119.63, 219.16]
Gestational diabetes, n (%)	
Yes	59 (7.37)
No	741 (92.62)

*Child characteristics, n* = *800*	
Sex, n (%)	
Male	403 (50.37)
Female	397 (49.62)
Birth weight (g)	3320 [3030, 3584]
Prematurity, n (%)	
Yes	28 (3.50)
No	772 (96.5)
Type of lactation at 15 days, n (%)	
Breastfeeding	571 (71.37)
Infant formula	45 (5.62)
Mixed	184 (23)
Delivery type, n (%)	
Caesarean	193 (24.12)
Vaginal	607 (75.87)

Data are expressed as median [IQR] for continuous variables, or number (percentage) for categorical variables

### Associations of maternal carbohydrate quantity and quality consumption with child BSID-III performance at 18 months of age

Increased carbohydrate consumption was inversely associated with language development and receptive language in both the crude (β coefficient (p-trend): − 2.59 (0.03), − 0.56 (0.02) respectively) and minimally adjusted models (β coefficient (p-trend): − 2.67 (0.03), − 0.58 (0.02) respectively). However, in the fully adjusted model, which included maternal and child-related variables, these associations were no longer significant, although the inverse direction of the association remained (Table 2). A sensitivity analysis that included maternal ethnicity revealed similar results, remaining non-significant (Tables S4). In a secondary analysis, replacing the type of lactation during the first 15 days of life with the type of lactation at 18 months of age did not affect the results, which remained non-statistically significant (Table S5).

**Table 2:** Associations between dietary carbohydrate intake during pregnancy and BSID-III infant performance at 18 months

Outcome model^2^	Tertiles of carbohydrate intake^1^			P trend	Carbohydrate intake^1^	*P*-value
	T1 (n = 202)	T2 (n = 202)	T3 (n = 191)		n = 595	
	146.86 [132.94, 161.84]	200.02 [188.40, 214.19]	272.56 [246.73, 307.34]		199.05 [161.29, 245.30]	
*Cognitive development*						
Model 1	Ref	1.61 (− 0.63, 3.85)	0.10 (− 2.17, 2.37)	0.967	8·10^−4^ (− 0.01, 0.01)	0.915
Model 2	Ref	1.46 (− 0.77, 3.70)	− 0.01 (− 2.28, 2.26)	0.898	1·10^−4^ (− 0.01, 0.01)	0.989
Model 3	Ref	0.76 (− 1.76, 3.28)	− 0.75 (− 4.41, 2.92)	0.658	− 2·10^−3^ (− 0.03, 0.03)	0.891
*Language development*						
Model 1	Ref	0.15 (− 2.27, 2.58)	− 2.59 (− 5.05, − 0.13)	0.030	− 0.01 (− 0.03, 4·10^−4^)	0.055
Model 2	Ref	0.03 (− 2.39, 2.45)	− 2.67 (− 5.13, − 0.21)	0.026	− 0.01 (− 0.03, 1·10^−4^)	0.051
Model 3	Ref	− 0.24 (− 2.95, 2.47)	− 2.31 (− 6.26, 1.64)	0.239	− 0.01 (− 0.04, 0.02)	0.567
*Expressive language*						
Model 1	Ref	0.03 (− 0.35, 0.42)	− 0.18 (− 0.57, 0.21)	0.328	− 1·10^−3^ (− 4·10^−3^, 1·10^−3^)	0.362
Model 2	Ref	0.01 (− 0.37, 0.40)	− 0.20 (− 0.58, 0.20)	0.291	− 1·10^−3^ (− 4·10^−3^, 1·10^−3^)	0.348
Model 3	Ref	− 0.01 (− 0.44, 0.42)	− 0.13 (− 0.76, 0.50)	0.666	− 3·10^−4^ (− 5·10^−3^, 5·10^−3^)	0.908
*Receptive language*						
Model 1	Ref	− 0.01 (− 0.50, 0.48)	− 0.56 (− 1.06, − 0.06)	0.022	− 3·10^−3^ (− 0.01, 1·10^−4^)	0.060
Model 2	Ref	− 0.04 (− 0.53, 0.44)	− 0.58 (− 1.07, − 0.08)	0.018	− 3·10^−3^ (− 0.01, 2·10^−5^)	0.051
Model 3	Ref	− 0.15 (− 0.69, 0.39)	− 0.59 (− 1.37, 0.20)	0.137	− 2·10^−3^ (− 0.01, 4·10^−3^)	0.480
*Motor development*						
Model 1	Ref	− 0.19 (− 1.83, 1.46)	− 0.60 (− 2.27, 1.07)	0.472	− 1·10^−3^ (− 0.01, 0.01)	0.822
Model 2	Ref	− 0.12 (− 1.77, 1.53)	− 0.54 (− 2.22, 1.13)	0.513	− 8·10^−4^ (− 0.01, 0.01)	0.881
Model 3	Ref	− 0.60 (− 2.48, 1.28)	− 0.87 (− 3.61, 1.87)	0.542	6·10^−3^ (− 0.01, 0.03)	0.559
*Fine motor*						
Model 1	Ref	0.22 (− 0.14, 0.57)	0.01 (− 0.35, 0.38)	0.974	3·10^−4^ (− 0.01, 0.01)	0.792
Model 2	Ref	0.21 (− 0.14, 0.57)	0.01 (− 0.35, 0.37)	0.955	3·10^−4^ (− 2·10^−3^, 3·10^−3^)	0.799
Model 3	Ref	0.04 (− 0.37, 0.46)	− 0.27 (− 0.87, 0.33)	0.364	− 9·10^−4^ (− 0.01, 4·10^−3^)	0.706
*Gross motor*						
Model 1	Ref	− 0.26 (− 0.59, 0.07)	− 0.25 (− 0.59, 0.08)	0.160	− 8·10^−4^ (− 3·10^−3^, 1·10^−3^)	0.450
Model 2	Ref	− 0.24 (− 0.56, 0.09)	− 0.23 (− 0.57, 0.10)	0.198	− 7·10^−4^ (− 3·10^−3^, 1·10^−3^)	0.521
Model 3	Ref	− 0.28 (− 0.65, 0.10)	− 0.20 (− 0.74, 0.35)	0.514	2·10^−3^ (− 2·10^−3^, 6·10^−3^)	0.346

^1^ Carbohydrates intake (g/day) is represented as median [IQR]

^2^ Results are expressed as β coefficients and 95% confidence interval estimated using linear model. Model 1: crude model; Model 2: adjusted for child sex and age at behavioral assessment and mother age at enrollment; Model 3: Model 2 + financial status, education, pregnancy body mass index, physical activity, pregnancy smoking status, adherence to Mediterranean diet (MEDAS), maternal energy intake and maternal gestational diabetes, child weight at birth, prematurity, type of lactation and type of delivery. Abbreviations: BSID-III, Bayley Scales of Infant and Toddler Development

Dietary GI was negatively associated with both language development and receptive language in the crude (β coefficient (p-trend): − 2.58 (0.04), − 0.53 (0.04) respectively) and minimally adjusted models (β coefficient (p-trend): − 2.73 (0.03), − 0.54 (0.04) respectively) (Table 3). However, the fully adjusted model and sensitivity analyses adjusting for maternal ethnicity or accounting for infant feeding type at 18 months showed that these associations were no longer statistically significant (Tables S4-5). In contrast, GI showed a consistent inverse association with gross motor development across crude, minimally and fully (β coefficient (p-trend): − 0.49 (0.01)) adjusted models (Table 3). These findings remained statistically significant in sensitivity analyses controlling for maternal ethnicity or infant feeding type at 18 months (Tables S4-5). Dietary GL was also negatively associated with language development and receptive language, although the association showed a non-significant trend (β coefficient (p-trend): − 3.13 (0.08), − 0.58 (0.11) respectively) in the fully adjusted model (Table 4). Additionally, adjusting for maternal ethnicity rendered the association non-significant (Table S4), while accounting for infant feeding at 18 months yielded similar results (Table S5).

**Table 3:** Associations between dietary GI during pregnancy and BSID-III infant performance at 18 months

Outcome model^2^	Tertiles of dietary GI^1^			P trend	Dietary GI^1^	*P*-value
	T1 (n = 200)	T2 (n = 201)	T3 (n = 194)		n = 595	
	39.19 [37.12, 40.80]	44.74 [43.65, 46.03]	53.27 [50.45, 58.14]		44.69 [40.78, 49.92]	
*Cognitive development*						
Model 1	Ref	0.35 (− 1.90, 2.59)	− 1.48 (− 3.75, 0.78)	0.160	− 0.05 (− 0.16, 0.07)	0.428
Model 2	Ref	0.37 (− 1.88, 2.61)	− 1.35 (− 3.64, 0.93)	0.200	− 0.04 (− 0.16, 0.08)	0.490
Model 3	Ref	0.61 (− 1.63, 2.58)	− 1.44 (− 3.76, 0.87)	0.173	− 0.03 (− 0.15, 0.09)	0.582
*Language development*						
Model 1	Ref	− 0.76 (− 3.20, 1.67)	− 2.58 (− 5.04, − 0.12)	0.036	− 0.09 (− 0.22, 0.33)	0.149
Model 2	Ref	− 0.88 (− 3.32, 1.56)	− 2.73 (− 5.21, − 0.26)	0.028	− 0.10 (− 0.23, 0.02)	0.111
Model 3	Ref	− 0.84 (− 3.26, 1.57)	− 2.36 (− 4.86, 0.14)	0.061	− 0.06 (− 0.19, 0.07)	0.366
*Expressive language*						
Model 1	Ref	0.08 (− 0.30, 0.46)	− 0.20 (− 0.59, 0.18)	0.252	− 0.01 (− 0.03, 0.01)	0.413
Model 2	Ref	0.05 (− 0.33, 0.43)	− 0.23 (− 0.61, 0.16)	0.213	− 0.01 (− 0.03, 0.01)	0.332
Model 3	Ref	0.04 (− 0.34, 0.43)	− 0.23 (− 0.63, 0.17)	0.220	− 0.01 (− 0.03, 0.01)	0.495
*Receptive language*						
Model 1	Ref	− 0.35 (− 0.84, 0.15)	− 0.53 (− 1.03, − 0.03)	0.043	− 0.02 (− 0.04, 0.01)	0.172
Model 2	Ref	− 0.35 (− 0.84, 0.14)	− 0.54 (− 1.04, − 0.04)	0.038	− 0.02 (− 0.04, 0.01)	0.144
Model 3	Ref	− 0.33 (− 0.81, 0.15)	− 0.41 (− 0.91, 0.08)	0.120	− 0.01 (− 0.03, 0.02)	0.601
*Motor development*						
Model 1	Ref	− 0.28 (− 1.93, 1.36)	− 1.42 (− 3.08, 0.24)	0.084	− 0.04 (− 0.12, 0.05)	0.393
Model 2	Ref	− 0.31 (− 1.96, 1.34)	− 1.57 (− 3.25, 0.10)	0.057	− 0.04 (− 0.13, 0.04)	0.313
Model 3	Ref	− 0.56 (− 2.24, 1.11)	− 1.51 (− 3.24, 0.22)	0.085	− 0.02 (− 0.11, 0.07)	0.624
*Fine motor*						
Model 1	Ref	0.15 (− 0.21, 0.51)	0.01 (− 0.35, 0.37)	0.964	3·10^−3^ (− 0.02, 0.02)	0.771
Model 2	Ref	0.14 (− 0.22, 0.50)	0.01 (− 0.36, 0.37)	0.955	3·10^−3^ (− 0.02, 0.02)	0.788
Model 3	Ref	0.14 (− 0.23, 0.50)	0.04 (− 0.34, 0.42)	0.881	0.01 (− 0.01, 0.03)	0.482
*Gross motor*						
Model 1	Ref	− 0.21 (− 0.54, 0.12)	− 0.43 (− 0.77, − 0.10)	0.011	− 0.02 (− 0.03, 9·10^−4^)	0.063
Model 2	Ref	− 0.21 (− 0.53, 0.12)	− 0.47 (− 0.80, − 0.14)	0.006	− 0.02 (− 0.03, − 7·10^−4^)	0.042
Model 3	Ref	− 0.29 (− 0.62, 0.05)	− 0.49 (− 0.84, − 0.15)	0.006	− 0.02 (− 0.03, 2·10^−3^)	0.078

^1^Dietary GI is represented as median [IQR]

^2^Results are expressed as β coefficients and 95% confidence interval estimated using linear model. Model 1: crude model; Model 2: adjusted for child sex and age at behavioral assessment and mother age at enrollment; Model 3: Model 2 + financial status, education, pregnancy body mass index, physical activity, pregnancy smoking status, adherence to Mediterranean diet (MEDAS), maternal energy intake and maternal gestational diabetes, child weight at birth, prematurity, type of lactation and type of delivery. Abbreviations: GI, glycemic index; BSID-III, Bayley Scales of Infant and Toddler Development

**Table 4:** Associations between dietary GL during pregnancy and BSID-III infant performance at 18 months

Outcome model^2^	Tertiles of dietary GL^1^			P trend	Dietary GL^1^	*P*-value
	T1 (n = 201)	T2 (n = 201)	T3 (n = 193)		n = 595	
	62.5 [53.5, 69.9]	91.1 [82.4, 97.7]	132 [118, 156]		90.84 [69.80, 116.73]	
*Cognitive development*						
Model 1	Ref	− 0.52 (− 2.77, 1.73)	− 0.81 (− 3.08, 1.46)	0.491	− 4·10^−3^ (− 0.03, 0.02)	0.725
Model 2	Ref	− 0.56 (− 2.81, 1.68)	− 0.84 (− 3.11, 1.42)	0.474	− 4·10^−3^ (− 0.03, 0.02)	0.724
Model 3	Ref	− 0.99 (− 3.46, 1.47)	− 1.58 (− 4.72, 1.56)	0.337	− 0.01 (− 0.05, 0.03)	0.579
*Language development*						
Model 1	Ref	− 1.84 (− 4.27, 0.59)	− 3.45 (− 5.91, − 1.00)	0.006	− 0.03 (− 0.06, − 5·10^−3^)	0.019
Model 2	Ref	− 1.93 (− 4.36, 0.49)	− 3.51 (− 5.96, − 1.07)	0.005	− 0.03 (− 0.06, − 0.01)	0.016
Model 3	Ref	− 1.95 (− 4.60, 0.71)	− 3.13 (− 6.48, 0.27)	0.080	− 0.02 (− 0.06, 0.01)	0.199
*Expressive language*						
Model 1	Ref	− 0.26 (− 0.64, 0.12)	− 0.36 (− 0.75, 0.03)	0.075	− 3·10^−3^ (− 0.01, 1·10^−3^)	0.193
Model 2	Ref	− 0.27 (− 0.66, 0.11)	− 0.37 (− 0.75, 0.01)	0.067	− 3·10^−3^ (− 0.01, 1·10^−3^)	0.169
Model 3	Ref	− 0.30 (− 0.73, 0.12)	− 0.42 (− 0.96, 0.11)	0.140	− 3·10^−3^ (− 0.01, 3·10^−3^)	0.378
*Receptive language*						
Model 1	Ref	− 0.39 (− 0.88, 0.10)	− 0.69 (− 1.19, − 0.19)	0.007	− 0.01 (− 0.01, − 6·10^−4^)	0.029
Model 2	Ref	− 0.41 (− 0.90, 0.08)	− 0.70 (− 1.20, − 0.21)	0.006	− 0.01 (− 0.01, − 8·10^−4^)	0.024
Model 3	Ref	− 0.43 (− 0.96, 0.10)	− 0.58 (− 1.25, 0.09)	0.107	− 4·10^−3^ (− 0.01, 4·10^−3^)	0.335
*Motor development*						
Model 1	Ref	− 0.45 (− 2.10, 1.20)	− 0.84 (− 2.50, 0.83)	0.328	− 3·10^−3^ (− 0.02, 0.01)	0.701
Model 2	Ref	− 0.45 (− 2.10, 1.20)	− 0.83 (− 2.50, 0.84)	0.332	− 4·10^−3^ (− 0.02, 0.01)	0.685
Model 3	Ref	− 0.66 (− 2.51, 1.18)	− 0.83 (− 3.18, 1.51)	0.511	− 3·10^−3^ (− 0.02, 0.03)	0.836
*Fine motor*						
Model 1	Ref	0.13 (− 0.23, 0.49)	− 0.01 (− 0.38, 0.35)	0.884	9·10^−4^ (− 3·10^−3^, 5·10^−3^)	0.642
Model 2	Ref	0.13 (− 0.23, 0.49)	− 0.13 (− 0.38, 0.35)	0.876	9·10^−4^ (− 3·10^−3^, 5·10^−3^)	0.653
Model 3	Ref	0.03 (− 0.37, 0.44)	− 0.12 (− 0.64, 0.39)	0.593	1·10^−3^ (− 5·10^−3^, 0.01)	0.694
*Gross motor*						
Model 1	Ref	− 0.29 (− 0.62, 0.04)	− 0.31 (− 0.64, 0.02)	0.082	− 2·10^−3^ (− 0.01, 1·10^−3^)	0.209
Model 2	Ref	− 0.28 (− 0.61, 0.05)	− 0.30 (− 0.64, 0.03)	0.086	− 2·10^−3^ (− 0.01, 1·10^−3^)	0.207
Model 3	Ref	− 0.29 (− 0.66, 0.07)	− 0.29 (− 0.76, 0.18)	0.271	− 1·10^−3^ (− 0.01, 4·10^−3^)	0.661

^1^Dietary GL is represented as median [IQR]

^2^Results are expressed as β coefficients and 95% confidence interval estimated using linear model. Model 1: crude model; Model 2: adjusted for child sex and age at behavioral assessment and mother age at enrollment; Model 3: Model 2 + financial status, education, pregnancy body mass index, physical activity, pregnancy smoking status, adherence to Mediterranean diet (MEDAS), maternal energy intake and maternal gestational diabetes, child weight at birth, prematurity, type of lactation and type of deliveryAbbreviations: GL, glycemic load; BSID-III, Bayley Scales of Infant and Toddler Development

Maternal CQI showed a positive association with gross motor development in all crude, minimally and fully (β coefficient (p-trend): 0.39 (0.01)) adjusted models (Table 5). This association remained statistically significant in sensitivity analysis that included maternal ethnicity (Table S4). However, when adjusting for infant feeding type at 18 months, similar positive trends were observed but did not reach statistical significance (β coefficient (p-trend): 0.31 (0.07)) (Table S5).

**Table 5:** Associations between dietary CQI during pregnancy and BSID-III infant performance at 18 months

Outcome model^2^	Tertiles of dietary CQI^1^			P trend	Dietary CQI^1^	*P*-value
	T1 (n = 193)	T2 (n = 134)	T3 (n = 268)		n = 595	
	11 [10, 12]	14 [14, 15]	16 [16, 17]		14 [12, 16]	
*Cognitive development*						
Model 1	Ref	0.76 (− 1.77, 3.29)	1.82 (− 0.30, 3.95)	0.111	0.28 (− 0.04, 0.61)	0.088
Model 2	Ref	0.42 (− 2.13, 2.97)	1.67 (− 0.47, 3.81)	0.162	0.26 (− 0.07, 0.59)	0.128
Model 3	Ref	0.49 (− 2.09, 3.07)	1.42 (− 0.81, 3.58)	0.247	0.25 (− 0.09, 0.59)	0.157
*Language development*						
Model 1	Ref	− 0.07 (− 2.82, 2.68)	0.86 (− 1.45, 3.17)	0.537	0.21 (− 0.15, 0.56)	0.249
Model 2	Ref	− 0.16 (− 2.93, 2.62)	0.92 (− 1.40, 3.25)	0.515	0.23 (− 0.13, 0.58)	0.217
Model 3	Ref	0.27 (− 2.51, 3.05)	1.37 (− 1.07, 3.67)	0.327	0.34 (− 0.03, 0.71)	0.074
*Expressive language*						
Model 1	Ref	0.02 (− 0.41, 0.45)	0.20 (− 0.17, 0.56)	0.345	0.02 (− 0.03, 0.08)	0.391
Model 2	Ref	0.02 (− 0.42, 0.45)	0.21 (− 0.15, 0.57)	0.318	0.03 (− 0.03, 0.08)	0.347
Model 3	Ref	0.12 (− 0.32, 0.57)	0.29 (− 0.09, 0.66)	0.162	0.05 (− 0.01, 0.10)	0.128
*Receptive language*						
Model 1	Ref	− 0.05 (− 0.60, 0.51)	0.18 (− 0.28, 0.65)	0.530	0.04 (− 0.03, 0.11)	0.302
Model 2	Ref	− 0.10 (− 0.66, 0.46)	0.18 (− 0.29, 0.65)	0.566	0.04 (− 0.03, 0.11)	0.301
Model 3	Ref	− 0.09 (− 0.64, 0.47)	0.21 (− 0.28, 0.67)	0.523	0.05 (− 0.03, 0.12)	0.208
*Motor development*						
Model 1	Ref	1.67 (− 0.18, 3.53)	0.82 (− 0.73, 2.38)	0.189	0.16 (− 0.07, 0.40)	0.176
Model 2	Ref	1.93 (0.05, 3.80)	0.99 (− 0.58, 2.56)	0.126	0.11 (− 0.05, 0.44)	0.113
Model 3	Ref	1.89 (− 0.03, 3.82)	0.88 (− 0.76, 2.52)	0.183	0.19 (− 0.06, 0.45)	0.139
*Fine motor*						
Model 1	Ref	0.19 (− 0.21, 0.60)	− 0.01 (− 0.35, 0.32)	0.879	(− 0.05, 0.06)	0.833
Model 2	Ref	0.20 (− 0.21, 0.60)	− 0.01 (− 0.35, 0.33)	0.877	(− 0.05, 0.06)	0.828
Model 3	Ref	0.12 (− 0.30, 0.55)	− 0.10 (− 0.45, 0.26)	0.746	− 0.01 (− 0.06, 0.05)	0.754
*Gross motor*						
Model 1	Ref	0.33 (− 0.04, 0.70)	0.29 (− 0.02, 0.60)	0.047	0.05 (1·10^−3^, 0.10)	0.045
Model 2	Ref	0.40 (0.03, 0.78)	0.33 (0.02, 0.65)	0.022	0.06 (0.01, 0.11)	0.020
Model 3	Ref	0.46 (0.08, 0.85)	0.39 (0.06, 0.71)	0.011	0.07 (0.02, 0.12)	0.005

^1^Dietary CQI is represented as median [IQR]

^2^Results are expressed as β coefficients and 95% confidence interval estimated using linear model. Model 1: crude model; Model 2: adjusted for child sex and age at behavioral assessment and mother age at enrollment; Model 3: Model 2 + financial status, education, pregnancy body mass index, physical activity, pregnancy smoking status, adherence to Mediterranean diet (MEDAS), maternal energy intake and maternal gestational diabetes, child weight at birth, prematurity, type of lactation and type of delivery. Abbreviations: CQI, carbohydrate quality index; BSID-III, Bayley Scales of Infant and Toddler Development

Overall, higher maternal GI and GL were associated with poorer language and receptive language outcomes in the crude and minimally adjusted models, while GI and CQI showed consistent negative and positive associations with gross motor development, respectively. These associations were attenuated or lost after adjusting for ethnicity or infant feeding at 18 months, although the direction generally remained.

### Associations of maternal carbohydrate quantity and quality consumption with child DP-3 performance at 8 and 28 months of age

A weak positive association between total carbohydrate intake and adaptive behaviour was observed in the crude and minimally adjusted models, but this association was no longer significant in the fully adjusted model (Table 6). In contrast, GI was negatively associated with motor development in both the minimally and fully adjusted models (β coefficient (p-trend): − 3.21 (0.01)), while the crude model showed only a tendency toward statistical significance (β coefficient (p-trend): − 2.20 (0.09)) (Table 7). This association remained after further adjustment for maternal ethnicity, or when considering the type of feeding at 18 months (Tables S6-7). No statistically significant associations were found between GL with child DP-3 performance (Tables 8).

**Table 6:** Associations between dietary carbohydrate intake during pregnancy and DP-3 infant performance at 8 and 28 months

Outcome model^2^	Tertiles of carbohydrate intake^1^			P trend	Carbohydrate intake^1^	*P*-value
	T1 (n = 100)	T2 (n = 119)	T3 (n = 107)		n = 326	
	141.72 [125.25, 157.67]	199.05 [188.78, 211.96]	270.62 [246.73, 296.08]		199.77 [162.51, 246.10]	
*Global Development Index*						
Model 1	Ref	1.52 (− 1.15, 4.19)	2.64 (− 0.09, 5.37)	0.063	0.01 (− 0.01, 0.03)	0.291
Model 2	Ref	1.73 (− 0.86, 4.32)	2.60 (− 0.05, 5.26)	0.064	0.01 (− 0.01, 0.03)	0.326
Model 3	Ref	0.51 (− 2.50, 3.53)	1.26 (− 2.94, 5.46)	0.562	− 0.02 (− 0.06, 0.01)	0.245
*Adaptive Behavior*						
Model 1	Ref	0.90 (− 1.29, 3.09)	2.32 (0.08, 4.57)	0.041	0.01 (− 5·10^−3^, 0.02)	0.196
Model 2	Ref	0.95 (− 1.17, 3.07)	2.26 (0.09, 4.43)	0.041	0.01 (− 5·10^−3^, 0.02)	0.232
Model 3	Ref	0.10 (− 2.38, 2.59)	1.85 (1.60, 5.30)	0.260	− 0.01 (− 0.03, 0.02)	0.684
*Social–Emotional*						
Model 1	Ref	1.54 (− 0.87, 3.96)	2.21 (− 0.27, 4.68)	0.093	0.01 (− 0.01, 0.02)	0.254
Model 2	Ref	1.46 (− 0.93, 3.84)	2.03 (− 0.41, 4.48)	0.119	0.01 (− 0.01, 0.02)	0.344
Model 3	Ref	0.80 (− 2.11, 3.50)	0.69 (− 3.30, 4.51)	0.809	− 0.02 (− 0.05, 0.02)	0.319
*Cognitive*						
Model 1	Ref	2.23 (− 0.21, 4.66)	2.08 (− 0.42, 4.58)	0.138	0.01 (− 0.01, 0.03)	0.206
Model 2	Ref	2.42 (− < 0.01, 4.84)	2.11 (− 0.37, 4.59)	0.140	0.01 (− 0.01, 0.03)	0.215
Model 3	Ref	0.83 (− 2.00, 3.66)	− 0.07 (− 4.01, 3.86)	0.886	− 0.02 (− 0.05, 0.01)	0.180
*Communication*						
Model 1	Ref	1.91 (− 0.91, 4.72)	1.71 (− 1.18, 4.60)	0.296	5·10^−3^ (− 0.01, 0.02)	0.621
Model 2	Ref	1.93 (− 0.71, 4.57)	1.50 (− 1.20, 4.20)	0.347	3·10^−3^ (− 0.01, 0.02)	0.761
Model 3	Ref	1.53 (− 1.64, 4.56)	0.83 (− 3.54, 5.08)	0.830	− 0.02 (− 0.06, 0.02)	0.292
*Motor development*						
Model 1	Ref	− 1.40 (− 4.09, 1.29)	0.84 (− 1.92, 3.59)	0.437	− 3·10^−3^ (− 0.02, 0.01)	0.776
Model 2	Ref	− 0.86 (− 3.27, 1.55)	1.10 (− 1.37, 3.57)	0.298	− 1·10^−3^ (− 0.02, 0.01)	0.897
Model 3	Ref	− 1.53 (− 4.23, 1.16)	1.12 (− 2.62, 4.87)	0.405	− 0.02 (− 0.05, 0.02)	0.358

^1^Carbohydrates intake (g/day) is represented as median [IQR]

^2^Results are expressed as β coefficients and 95% confidence interval estimated using linear mixed-effects model. Model 1: crude model; Model 2: adjusted for child sex and age at behavioral assessment and mother age at enrollment; Model 3: Model 2 + financial status, education, pregnancy body mass index, physical activity, pregnancy smoking status, adherence to Mediterranean diet (MEDAS), maternal energy intake and maternal gestational diabetes, child weight at birth, prematurity, type of lactation and type of delivery. Abbreviations: DP3, Developmental Profile 3

**Table 7:** Associations between dietary GI during pregnancy and DP-3 infant performance at 8 and 28 months

Outcome model^2^	Tertiles of dietary GI^1^			P trend	Dietary GI^1^	*P*-value
	T1 (n = 116)	T2 (n = 104)	T3 (n = 106)		n = 326	
	39.40 [37.27, 41.26]	44.73 [43.62, 45.83]	53.60 [50.49, 58.08]		44.65 [40.92, 50.45]	
*Global Development Index*						
Model 1	Ref	0.55 (− 2.11, 3.22)	− 0.18 (− 2.83, 2.48)	0.853	− 0.03 (− 0.17, 0.11)	0.688
Model 2	Ref	0.86 (− 1.71, 3.43)	− 1.00 (− 3.57, 1.57)	0.391	− 0.07 (− 0.21, 0.06)	0.277
Model 3	Ref	0.97 (− 1.65, 3.44)	− 1.77 (− 4.05, 1.28)	0.279	− 0.07 (− 0.22, 0.07)	0.313
*Adaptive Behavior*						
Model 1	Ref	0.57 (− 1.62, 2.77)	− 0.19 (− 2.37, 2.00)	0.814	− 0.05 (− 0.16, 0.07)	0.434
Model 2	Ref	0.91 (− 1.19, 3.02)	− 0.85 (− 2.95, 1.26)	0.367	− 0.09 (− 0.20, 0.02)	0.128
Model 3	Ref	0.78 (− 1.32, 2.88)	− 1.18 (− 3.38, 1.01)	0.262	− 0.09 (− 0.20, 0.03)	0.153
*Social–Emotional*						
Model 1	Ref	0.65 (− 1.77, 3.06)	0.29 (− 2.11, 2.70)	0.851	− 0.01 (− 0.13, 0.12)	0.891
Model 2	Ref	0.85 (− 1.52, 3.22)	− 0.25 (− 2.62, 2.11)	0.772	− 0.04 (− 0.17, 0.08)	0.492
Model 3	Ref	0.61 (− 1.44, 3.31)	− 0.92 (− 2.84, 2.12)	0.727	− 0.04 (− 0.17, 0.09)	0.534
*Cognitive*						
Model 1	Ref	0.79 (− 1.65, 3.22)	0.95 (− 1.48, 3.38)	0.466	0.06 (− 0.07, 0.18)	0.372
Model 2	Ref	0.93 (− 1.48, 3.34)	0.51 (− 1.90, 2.92)	0.724	0.03 (− 0.09, 0.16)	0.593
Model 3	Ref	1.02 (− 1.60, 3.19)	− 0.83 (− 2.79, 2.22)	0.779	0.01 (− 0.12, 0.14)	0.884
*Communication*						
Model 1	Ref	0.57 (− 2.24, 3.38)	0.38 (− 2.42, 3.18)	0.816	0.01 (− 0.14, 0.15)	0.933
Model 2	Ref	0.67 (− 1.95, 3.30)	− 0.33 (− 2.95, 2.29)	0.759	− 0.03 (− 0.17, 0.10)	0.618
Model 3	Ref	1.25 (− 1.43, 3.82)	− 0.44 (− 2.87, 2.61)	0.878	− 0.01 (− 0.16, 0.13)	0.840
*Motor development*						
Model 1	Ref	− 0.29 (− 2.97, 2.37)	− 2.20 (− 4.86, 0.46)	0.095	− 0.11 (− 0.25, 0.03)	0.113
Model 2	Ref	− 0.01 (− 2.38, 2.37)	− 2.76 (− 5.13, − 0.39)	0.018	− 0.14 (− 0.26, − 0.01)	0.031
Model 3	Ref	− 0.19 (− 2.47, 2.07)	− 3.21 (− 5.50, − 0.76)	0.010	− 0.13 (− 0.26, − 4·10^−3^)	0.044

^1^Dietary GI is represented as median [IQR]

^2^Results are expressed as β coefficients and 95% confidence interval estimated using linear mixed-effects model. Model 1: crude model; Model 2: adjusted for child sex and age at behavioral assessment and mother age at enrollment; Model 3: Model 2 + financial status, education, pregnancy body mass index, physical activity, pregnancy smoking status, adherence to Mediterranean diet (MEDAS), maternal energy intake and maternal gestational diabetes, child weight at birth, prematurity, type of lactation and type of delivery. Abbreviations: GI, glycemic index; DP3, Developmental Profile 3

**Table 8:** Associations between dietary GL during pregnancy and DP-3 infant performance at 8 and 28 months

Outcome model^2^	Tertiles of dietary GL^1^			P trend	Dietary GL^1^	*P*-value
	T1 (n = 108)	T2 (n = 109)	T3 (n = 109)		n = 326	
	60.1 [51.4, 69.3]	91.1 [82.4, 97.0]	132 [118, 154]		91.09 [69.28, 117.79]	
*Global Development Index*						
Model 1	Ref	3.34 (0.68, 6.00)	1.69 (− 0.97, 4.35)	0.304	0.01 (− 0.02, 0.03)	0.680
Model 2	Ref	3.44 (0.87, 6.01)	1.15 (− 1.42, 3.73)	0.535	1·10^−4^ (− 0.3, 0.03)	0.994
Model 3	Ref	1.86 (− 1.04, 4.70)	− 1.18 (− 4.82, 2.42)	0.382	− 0.03 (− 0.08, 0.01)	0.106
*Adaptive Behavior*						
Model 1	Ref	2.74 (0.56, 4.93)	1.13 (− 1.05, 3.32)	0.431	0.01 (− 0.02, 0.03)	0.637
Model 2	Ref	2.81 (0.70, 4.91)	0.64 (− 1.46, 2.75)	0.740	2·10^−4^ (− 0.02, 0.02)	0.988
Model 3	Ref	1.33 (− 1.04, 3.69)	− 1.40 (− 4.39, 1.58)	0.255	− 0.02 (− 0.06, 0.01)	0.162
*Social–Emotional*						
Model 1	Ref	3.59 (1.19, 5.99)	1.32 (− 1.08, 3.72)	0.425	0.01 (− 0.02, 0.03)	0.621
Model 2	Ref	3.54 (1.19, 5.90)	0.85 (− 1.51, 3.21)	0.686	9·10^−4^ (− 0.02, 0.03)	0.943
Model 3	Ref	2.38 (− 0.32, 5.00)	− 1.20 (− 4.59, 2.12)	0.309	− 0.03 (− 0.07, 0.01)	0.151
*Cognitive*						
Model 1	Ref	3.87 (1.45, 6.28)	2.18 (− 0.23, 4.60)	0.136	0.02 (− 0.01, 0.04)	0.236
Model 2	Ref	3.96 (1.57, 6.36)	1.93 (− 0.47, 4.33)	0.204	0.01 (− 0.01, 0.04)	0.324
Model 3	Ref	2.67 (0.01, 5.35)	− 0.33 (− 3.71, 3.05)	0.620	− 0.02 (− 0.06, 0.02)	0.381
*Communication*						
Model 1	Ref	1.49 (− 1.33, 4.31)	1.07 (− 1.75, 3.90)	0.504	4·10^−3^ (− 0.03, 0.03)	0.786
Model 2	Ref	1.40 (− 1.23, 4.04)	0.52 (− 2.12, 3.17)	0.772	− 2·10^−3^ (− 0.03, 0.03)	0.879
Model 3	Ref	0.70 (− 2.28, 3.66)	− 0.35 (− 4.10, 3.40)	0.792	− 0.02 (− 0.06, 0.02)	0.345
*Motor development*						
Model 1	Ref	− 0.22 (− 2.91, 2.47)	0.13 (− 2.56, 2.83)	0.905	− 0.01 (− 0.04, 0.01)	0.350
Model 2	Ref	0.09 (− 2.32, 2.51)	− 0.03 (− 2.45, 2.39)	0.976	− 0.01 (− 0.04, 0.01)	0.265
Model 3	Ref	− 0.91 (− 3.50, 1.69)	− 1.03 (− 4.30, 2.25)	0.583	− 0.04 (− 0.07, 2·10^−3^)	0.066

^1^Dietary GL is represented as median [IQR]

^2^Results are expressed as β coefficients and 95% confidence interval estimated using linear mixed-effects model. Model 1: crude model; Model 2: adjusted for child sex and age at behavioral assessment and mother age at enrollment; Model 3: Model 2 + financial status, education, pregnancy body mass index, physical activity, pregnancy smoking status, adherence to Mediterranean diet (MEDAS), maternal energy intake and maternal gestational diabetes, child weight at birth, prematurity, type of lactation and type of deliveryAbbreviations: GL, glycemic load; DP3, Developmental Profile 3

Similarly, CQI was positively associated with motor development in the minimally adjusted model (β coefficient (p-trend): 2.29 (0.04)) (Table 9). Sensitivity analysis further adjusting for maternal ethnicity showed similar significant positive associations, whereas adjustment for infant feeding type at 18 months showed a positive but non-statistically significant association (β coefficient (p-trend): 2.30 (0.06)) (Tables S6-7).

**Table 9:** Associations between dietary CQI during pregnancy and DP-3 infant performance at 8 and 28 months

Outcome model^2^	Tertiles dietary CQI^1^			P trend	Dietary CQI^1^	*P*-value
	T1 (n = 99)	T2 (n = 75)	T3 (n = 152)		n = 326	
	11 [10, 12]	14 [14, 15]	16 [16, 17]		14 [12, 16]	
*Global Development Index*						
Model 1	Ref	0.08 (− 2.95, 3.10)	0.62 (− 1.93, 3.17)	0.669	0.19 (− 0.20, 0.57)	0.337
Model 2	Ref	0.94 (− 2.00, 3.88)	1.55 (− 0.93, 4.03)	0.231	0.33 (− 0.04, 0.71)	0.085
Model 3	Ref	0.69 (− 2.31, 3.69)	1.05 (− 1.54, 3.65)	0.443	0.23 (− 0.18, 0.63)	0.271
*Adaptive Behavior*						
Model 1	Ref	− 0.13 (− 2.61, 2.35)	0.88 (− 1.21, 2.97)	0.488	0.14 (− 0.17, 0.46)	0.377
Model 2	Ref	0.66 (− 1.75, 3.06)	1.62 (− 0.41, 3.64)	0.142	0.26 (− 0.05, 0.57)	0.098
Model 3	Ref	0.75 (− 1.72, 3.22)	1.27 (− 0.87, 3.41)	0.267	0.18 (− 0.15, 0.52)	0.279
*Social–Emotional*						
Model 1	Ref	− 1.28 (− 4.01, 1.46)	− 0.50 (− 2.81, 1.81)	0.568	0.09 (− 0.26, 0.44)	0.630
Model 2	Ref	0.81 (− 3.52, 1.90)	− 0.02 (− 2.31, 2.26)	0.889	0.17 (− 0.17, 0.52)	0.326
Model 3	Ref	− 1.56 (− 4.35, 1.22)	− 0.85 (− 3.27, 1.56)	0.415	0.04 (− 0.34, 0.42)	0.838
*Cognitive*						
Model 1	Ref	− 0.75 (− 3.51, 2.01)	3·10^−3^ (− 2.33, 2.33)	0.910	0.04 (− 0.32, 0.39)	0.837
Model 2	Ref	− 0.31 (− 3.06, 2.45)	0.51 (− 1.81, 2.84)	0.739	0.11 (− 0.24, 0.47)	0.529
Model 3	Ref	0.13 (− 3.03, 2.59)	0.51 (− 2.12, 2.75)	0.857	0.09 (− 0.29, 0.47)	0.632
*Communication*						
Model 1	Ref	1.31 (− 1.87, 4.50)	0.29 (− 2.39, 2.98)	0.718	0.10 (− 0.30, 0.51)	0.618
Model 2	Ref	1.81 (− 1.18, 4.80)	0.96 (− 1.56, 3.49)	0.370	0.21 (− 0.17, 0.59)	0.284
Model 3	Ref	1.45 (− 1.63, 4.54)	0.70 (− 1.97, 3.37)	0.528	0.13 (− 0.28, 0.55)	0.534
*Motor development*						
Model 1	Ref	1.12 (− 1.91, 4.16)	1.42 (− 1.14, 3.98)	0.269	0.30 (− 0.09, 0.68)	0.133
Model 2	Ref	1.94 (− 0.78, 4.66)	2.29 (− 3·10^−3^, 4.59)	0.047	0.41 (0.06, 0.76)	0.022
Model 3	Ref	2.12 (− 0.56, 4.80)	2.22 (− 0.10, 4.54)	0.057	0.37 (0.01, 0.73)	0.047

^1^Dietary CQI is represented as median [IQR]

^2^Results are expressed as β coefficients and 95% confidence interval estimated using linear mixed-effects model. Model 1: crude mode; Model 2: adjusted for child sex and age at behavioral assessment and mother age at enrollment; Model 3: Model 2 + financial status, education, pregnancy body mass index, physical activity, pregnancy smoking status, adherence to Mediterranean diet (MEDAS), maternal energy intake and maternal gestational diabetes, child weight at birth, prematurity, type of lactation and type of delivery. Abbreviations: CQI, carbohydrate quality index; DP3, Developmental Profile 3

Considering all results together, GI showed consistent negative associations with childhood motor development, while CQI showed this association only in the minimally adjusted model. Total carbohydrate intake and GL were not associated with any outcome.

## Discussion

In this prospective cohort study, increased maternal dietary glycemic index (GI) and glycemic load (GL) during pregnancy were associated with lower language and receptive language scores in children at 18 months, as measured by the BSID-III, in the crude and minimally adjusted models. In addition, higher maternal GI and lower carbohydrate quality index (CQI) were consistently linked to reduced motor development scores, as assessed by the BSID-III at 18 months and the DP-3 at 8 and 28 months. These associations remained robust across adjusted models, suggesting a sustained influence of maternal dietary glycemic quality on motor developmental trajectories in early childhood.

The receptive communication subscale of the BSID-III assesses a broad range of early communication skills, including preverbal behavior, vocabulary acquisition, social referencing, and verbal comprehension. Lower scores in this subscale indicate difficulties in fundamental developmental areas such as recognizing familiar words, identifying objects, understanding spoken instructions, following directions, and understanding fundamental concepts [31]. Thus, the observed associations between GI, GL and language outcomes on the BSID-III scale may reflect early challenges in critical cognitive and communicative abilities influenced by maternal dietary carbohydrate quality during pregnancy. The gross motor subscale of the BSID-III evaluates several aspects of early movement and play, including head control, rolling, sitting, walking and balance, all fundamental for further neuromotor and cognitive development [31]. Similarly, the motor domain from the DP-3 assesses muscle coordination, strength, endurance, flexibility, and the execution of sequential motor tasks [27]. The observed negative association between GI and the positive association of CQI with motor development scores highlights the clinical relevance of these findings as impaired motor development at these early stages may indicate delayed neuromuscular maturation and could predict longer-term developmental challenges. Our findings are supported by previous research. A prospective cohort study published in 2024 identified a positive association between maternal dietary GI and the risk of neurodevelopmental delay in children [32]. Furthermore, the maternal consumption of moderate to high GI food during pregnancy, such as sucrose, sugar-sweetened beverages, low consumption of wholefoods and high processed diets, has been associated with poorer problem-solving abilities, deficits in verbal and visual memory, and impaired learning capacities, affecting both verbal knowledge and non-verbal skills [8, 32, 33]. In contrast, the consumption of fiber during pregnancy, a known modulator of postprandial glycemic response, reduced the risk of neurodevelopmental delay and improved the motor performance at 5–6 years old [9, 34]. Together, these findings highlight the relevance of our results, suggesting that early motor delays observed in our cohort may reflect slower neuromuscular development influenced by maternal dietary patterns during pregnancy.

One potential biological mechanism underlying these associations could involve the insulin response required to manage higher postprandial glycemia after consuming high-GI and high-GL foods. Chronic exposure to elevated glucose and insulin levels can trigger systemic inflammation, which may affect fetal brain and immune system development through epigenetic changes and inflammatory pathways, particularly during critical periods of central nervous system, microglial and immune maturation [35–37]. Maternal conditions characterized by systemic inflammation and insulin resistance, such as pre-pregnancy overweight and gestational diabetes, have been associated to an increased risk of neurodevelopmental delays in offspring [38–40]. Additionally, compared to high-GI/GL foods, low-GI/GL foods typically have a healthier nutritional profile, being richer in vitamins and minerals. These micronutrient differences could also support fetal brain development and contribute to improved health outcomes [5, 41]. In this context, higher dietary CQI has been associated with lower risk of inadequacy in meeting micronutrient recommendations [15]. However, in our study, the associations between higher maternal GI and lower CQI with lower child neurodevelopment scores remained statistically significant even after adjusting for adherence to a Mediterranean dietary pattern, which is itself typically rich in micronutrients. This suggests that the benefits extend beyond simply adhering to a generally healthy diet and highlights that GI, GL and CQI can serve as useful indicators of maternal intake of nutrient-dense foods that promote optimal fetal brain development.

Regarding total carbohydrate intake, we found no beneficial or adverse associations with childhood neurodevelopmental outcomes. The Recommended Dietary Allowance (RDA) for carbohydrates, as established by the institute of Medicine (IOM) Food and Nutrition Board, is set at ≥ 175 g/d or 45–65% of total energy intake [42]. In the BiSC population, the median carbohydrate intake was approximately 200 g/d, which meet these recommendations but was at the lower range of the recommended percentage intake. Taken together, our findings suggest that when carbohydrate intake is within the recommended range, total quantity alone may not significantly influence in neurodevelopmental outcomes. However, our results emphasize the importance of managing GI, GL and CQI during pregnancy, even if carbohydrate intake is at the lower end of the recommended levels. Prioritizing low-GI/GL and high-CQI carbohydrate sources, such as whole grains, pulses, legumes, fruits, and nuts, over refined grains, added sugars, and sugar-sweetened beverages, may help to promote child neurodevelopment.

This study has several strengths, including its prospective design and a robust sample size. Moreover, the comprehensive assessment of maternal diet and lifestyle using validated questionnaires enhances the reliability and accuracy of the results. However, certain limitations should be acknowledged. First, neurodevelopmental assessments were limited to the first 28 months of life, and longer follow-up periods would be valuable for evaluating the long-term associations of maternal dietary patterns on child neurodevelopment. Furthermore, parental reporting of DP-3 could potentially induce a bias. Second, although FFQ provide a valuable tool to comprehensively assess dietary patterns, they remain prone to recall bias and misreporting; and nutrient estimations also rely on food composition tables, which may not fully capture variability in nutrient content. Furthermore, as an observational study, causality cannot be established, and the biological mechanisms underlying the observed associations remain unclear. Another relevant limitation of this study is the potential influence of residual confounding. Although extensive adjustment for a wide range of maternal, perinatal, and sociodemographic variables did not substantially alter the direction of the associations, in some cases, particularly after further adjusting for maternal ethnicity and the type of infant feeding at 18 months of age, the associations lost statistical significance. The uneven distribution of participants across ethnicity categories may partly explain this attenuation. Furthermore, the subset of participants with available information on the type of infant lactation at 18 months of age was limited. All these factors could lead to a reduction of statistical power and stability of the estimates. Although multiple testing can increase the risk of Type I error, our analyses were limited to pre-specified hypotheses, and reporting uncorrected results allows the observed effect sizes and confidence intervals to be fully appreciated. Sensitivity analyses confirmed that the main findings are robust, though marginally significant associations should still be interpreted with caution. Future research involving larger and more diverse cohorts will be essential to validate these findings and to better clarify the biological mechanisms underlying the relationship between maternal carbohydrate quality and child neurodevelopment.

In conclusion, our study provides novel evidence showing that the quality of maternal dietary carbohydrates during pregnancy, particularly diets high in GI and GL and low in CQI, is associated with poorer offspring development, especially with lower motor, and to a lesser extent, language development scores in offspring. These findings highlight the importance of incorporating dietary counselling into prenatal care, emphasizing not only the quantity but especially the quality of carbohydrates. Such measures, combined with structured nutritional follow-up and evidence-based policy recommendations, such guidance can serve as practical strategy to support optimal child neurodevelopmental outcomes. However, given the small differences in beta coefficients and the attenuation of some associations after adjusting for socio-demographic factors such as ethnicity, further studies in larger and more diverse cohorts are needed to confirm these findings across multicultural and ethnically varied populations. Overall, our results represent a significant public health opportunity to improve child neurocognitive outcomes by prioritizing high-quality carbohydrate sources with low GI, low GL, and high CQI during pregnancy.

## Supplementary Information

Below is the link to the electronic supplementary material.

Supplementary file1 (DOCX 122 KB)

Figure S1. Food groups contribution to maternal A) total carbohydrate intake, B) dietary glycemic index and C) dietary glycemic load during pregnancy. Supplementary file2 (PDF 101 KB)

## Data Availability

Data will be made available upon reasonable request.

## References

[1] Cusick SE, Georgieff MK (2016) The role of nutrition in brain development: the golden opportunity of the “first 1000 days.” J Pediatr 175:16. 10.1016/J.JPEDS.2016.05.013 27266965 10.1016/j.jpeds.2016.05.013 PMC4981537

[2] Cortés-Albornoz MC, García-Guáqueta DP, Velez-Van-meerbeke A, Talero-Gutiérrez C (2021) Maternal nutrition and neurodevelopment: a scoping review. Nutrients 13:3530. 10.3390/NU13103530 34684531 10.3390/nu13103530 PMC8538181

[3] Parisi F, Laoreti A, Cetin I (2014) Multiple micronutrient needs in pregnancy in industrialized countries. Ann Nutr Metab 65:13–21. 10.1159/000365794 25227491 10.1159/000365794

[4] Plećaš D, Plešinac S, Vučinić OK (2014) Nutrition in pregnancy: basic principles and recommendations. Srp Arh Celok Lek 142:125–130. 10.2298/SARH1402125P 24684045 10.2298/sarh1402125p

[5] Crovetto F, Nakaki A, Arranz A, Borras R, Vellvé K, Paules C et al (2023) Effect of a Mediterranean diet or mindfulness-based stress reduction during pregnancy on child neurodevelopment: a prespecified analysis of the IMPACT BCN randomized clinical trial. JAMA Netw Open 6:e2330255. 10.1001/JAMANETWORKOPEN.2023.30255 37606923 10.1001/jamanetworkopen.2023.30255 PMC10445211

[6] Ouyang J, Cai W, Wu P, Tong J, Gao G, Yan S et al (2024) Association between dietary patterns during pregnancy and children’s neurodevelopment: a birth cohort study. Nutrients. 10.3390/NU16101530 38794768 10.3390/nu16101530 PMC11123670

[7] Dai FC, Wang P, Li Q, Zhang L, Yu LJ, Wu L et al (2023) Mediterranean diet during pregnancy and infant neurodevelopment: a prospective birth cohort study. Front Nutr. 10.3389/FNUT.2022.1078481 36726814 10.3389/fnut.2022.1078481 PMC9885498

[8] Gamba RJ, Leung CW, Petito L, Abrams B, Laraia BA (2019) Sugar sweetened beverage consumption during pregnancy is associated with lower diet quality and greater total energy intake. PLoS ONE 14:e0215686. 10.1371/JOURNAL.PONE.0215686 31022225 10.1371/journal.pone.0215686 PMC6483237

[9] Miyake K, Horiuchi S, Shinohara R, Kushima M, Otawa S, Yui H et al (2023) Maternal dietary fiber intake during pregnancy and child development: the Japan environment and children’s study. Front Nutr 10:1203669. 10.3389/FNUT.2023.1203669 37575329 10.3389/fnut.2023.1203669 PMC10415901

[10] Bordeleau M, Fernández de Cossío L, Chakravarty MM, Tremblay MÈ (2021) From maternal diet to neurodevelopmental disorders: a story of neuroinflammation. Front Cell Neurosci 14:612705. 10.3389/FNCEL.2020.612705 33536875 10.3389/fncel.2020.612705 PMC7849357

[11] Dienel GA (2019) Brain glucose metabolism: Integration of energetics with function. Physiol Rev 99:949–1045. 10.1152/PHYSREV.00062.2017/ASSET/IMAGES/LARGE/Z9J0011928950009.JPEG 30565508 10.1152/physrev.00062.2017

[12] Sünram-Lea SI, Owen L (2017) The impact of diet-based glycaemic response and glucose regulation on cognition: evidence across the lifespan. Proc Nutr Soc 76:466–477. 10.1017/S0029665117000829 28651658 10.1017/S0029665117000829

[13] Liu C, Meng Q, Zu C, Wei Y, Su X, Zhang Y et al (2023) Dietary low- and high-quality carbohydrate intake and cognitive decline: a prospective cohort study in older adults. Clin Nutr 42:1322–1329. 10.1016/J.CLNU.2023.06.021 37413810 10.1016/j.clnu.2023.06.021

[14] Jenkins DJ, Willett WC (2024) Perspective on the health value of carbohydrate-rich foods: glycemic index and load; fiber and whole grains. Am J Clin Nutr 120:468–470. 10.1016/J.AJCNUT.2024.07.004 39232600 10.1016/j.ajcnut.2024.07.004 PMC11393399

[15] Zazpe I, Sánchez-Taínta A, Santiago S, De La Fuente-Arrillaga C, Bes-Rastrollo M, Martínez JA et al (2014) Association between dietary carbohydrate intake quality and micronutrient intake adequacy in a Mediterranean cohort: the SUN (Seguimiento Universidad de Navarra) project. Br J Nutr 111:2000–2009. 10.1017/S0007114513004364 24666554 10.1017/S0007114513004364

[16] Dadvand P, Gascon M, Bustamante M, Rivas I, Foraster M, Basagaña X et al (2024) Cohort profile: Barcelona life study cohort (BiSC). Int J Epidemiol 53:63. 10.1093/IJE/DYAE063 10.1093/ije/dyae063 38725298

[17] Liu S, Willett WC, Stampfer MJ, Hu FB, Franz M, Sampson L et al (2000) A prospective study of dietary glycemic load, carbohydrate intake, and risk of coronary heart disease in US women. Am J Clin Nutr 71:1455–1461. 10.1093/AJCN/71.6.1455 10837285 10.1093/ajcn/71.6.1455

[18] Vioque J, Navarrete-Muñoz EM, Gimenez-Monzó D, García-De-La-Hera M, Granado F, Young IS et al (2013) Reproducibility and validity of a food frequency questionnaire among pregnant women in a Mediterranean area. Nutr J 12:26. 10.1186/1475-2891-12-26 23421854 10.1186/1475-2891-12-26 PMC3584829

[19] Moreiras O, Carbajal A, Cabrera L (1992) Food composition tables. Ediciones, Pirámide, SA

[20] Farran A, Zamora R, Cervera P (2003) CESNID food composition tables

[21] BEDCA Network of the Spanish Ministry of Science and Innovation (2010) Spanish Food Composition Database (BEDCA)

[22] Atkinson FS, Brand-Miller JC, Foster-Powell K, Buyken AE, Goletzke J (2021) International tables of glycemic index and glycemic load values 2021: a systematic review. Am J Clin Nutr 114:1625–1632. 10.1093/AJCN/NQAB233 34258626 10.1093/ajcn/nqab233

[23] Louie JCY, Flood V, Turner N, Everingham C, Gwynn J (2011) Methodology for adding glycemic index values to 24-hour recalls. Nutrition 27:59–64. 10.1016/j.nut.2009.12.006 20541365 10.1016/j.nut.2009.12.006

[24] Bayley N (2015) Bayley-III. Escalas Bayley de Desarrollo Infantil. Pearson, Madrid

[25] Alpern Gerald D (2018) DP-3: Perfil de desarrollo-3. Adaptación española: Sánchez-Sánchez F. TEA Ediciones, Madrid

[26] Case-Smith J, Alexander H (2010) The Bayley-III motor scale. In: Weiss LG, Oakland T, Aylward GP (eds) Bayley-III clinical use and interpretation. Elsevier, Amsterdam, pp 77–146. 10.1016/B978-0-12-374177-6.10004-2

[27] Alpern GD (2007) Developmental profile 3: DP-3. Western Psychological Services (WPS), Torrance, CA

[28] Alesi M, Giustino V, Gentile A, Gómez-López M, Battaglia G (2022) Motor coordination and global development in subjects with Down syndrome: the influence of physical activity. J Clin Med 11:5031. 10.3390/JCM11175031 36078962 10.3390/jcm11175031 PMC9457525

[29] García-Conesa MT, Philippou E, Pafilas C, Massaro M, Quarta S, Andrade V et al (2020) Exploring the validity of the 14-item Mediterranean Diet Adherence Screener (MEDAS): a cross-national study in seven European countries around the Mediterranean region. Nutrients 12:1–18. 10.3390/NU12102960 10.3390/nu12102960 PMC7601687 32992649

[30] Chasan-taber L, Schmidt MD, Roberts DE, Hosmer D, Markenson G, Freedson PS et al (2004) Development and validation of a pregnancy physical activity questionnaire. Med Sci Sports Exerc 36:1750–1760. 10.1249/01.MSS.0000142303.49306.0D 15595297 10.1249/01.mss.0000142303.49306.0d

[31] Michalec D (2011) Bayley scales of infant development: third edition. In: Goldstein S, Naglieri JA (eds) Encyclopedia of child behavior and development. Springer, Berlin, p 215. 10.1007/978-0-387-79061-9_295

[32] Gogos A, Thomson S, Drummond K, Holland L, O’Hely M, Dawson S et al (2024) Socioeconomic adversity, maternal nutrition, and the prenatal programming of offspring cognition and language at two years of age through maternal inflammation. Brain Behav Immun 122:471–482. 10.1016/J.BBI.2024.08.033 39163911 10.1016/j.bbi.2024.08.033

[33] Cohen JFW, Rifas-Shiman SL, Young J, Oken E (2018) Associations of prenatal and child sugar intake with child cognition. Am J Prev Med 54:727–735. 10.1016/J.AMEPRE.2018.02.020 29674185 10.1016/j.amepre.2018.02.020 PMC5962431

[34] Saros L, Setänen S, Hieta J, Kataja EL, Suorsa K, Vahlberg T et al (2025) The effect of maternal risk factors during pregnancy on children’s motor development at 5–6 years. Clin Nutr ESPEN 66:236–244. 10.1016/J.CLNESP.2025.01.047 39870192 10.1016/j.clnesp.2025.01.047

[35] Han VX, Patel S, Jones HF, Dale RC (2021) Maternal immune activation and neuroinflammation in human neurodevelopmental disorders. Nat Rev Neurol 17(9):564–579. 10.1038/s41582-021-00530-8 34341569 10.1038/s41582-021-00530-8

[36] Wang H, Zu P, Yin W, Zhang L, Ruan L, Chen X et al (2025) Maternal insulinemic and inflammatory dietary patterns and risk of child neurodevelopmental delay. Eur J Nutr 64:1–9. 10.1007/S00394-024-03531-7/FIGURES/2 10.1007/s00394-024-03531-7 39589432

[37] Wang H, Yin W, Ma S, Wang P, Zhang L, Li P et al (2024) Prenatal environmental adversity and child neurodevelopmental delay: the role of maternal low-grade systemic inflammation and maternal anti-inflammatory diet. Eur Child Adolesc Psychiatry 33:1771–1781. 10.1007/S00787-023-02267-9/METRICS 37596369 10.1007/s00787-023-02267-9

[38] Perea V, Urquizu X, Valverde M, Macias M, Carmona A, Esteve E et al (2022) Influence of maternal diabetes on the risk of neurodevelopmental disorders in offspring in the prenatal and postnatal periods. Diabetes Metab J 46:912–922. 10.4093/DMJ.2021.0340 35488357 10.4093/dmj.2021.0340 PMC9723192

[39] Nivins S, Giesbrecht GF, Tomfohr-Madsen L, Lebel C (2024) Prenatal maternal diabetes, comorbidities, and risk for neurodevelopmental impairment in the first two years. Pediatr Res. 10.1038/s41390-024-03620-7 39390101 10.1038/s41390-024-03620-7

[40] Gumusoglu SB, Stevens HE (2019) Maternal inflammation and neurodevelopmental programming: a review of preclinical outcomes and implications for translational psychiatry. Biol Psychiatry 85:107–121. 10.1016/J.BIOPSYCH.2018.08.008 30318336 10.1016/j.biopsych.2018.08.008

[41] Puig-Vallverdú J, Romaguera D, Fernández-Barrés S, Gignac F, Ibarluzea J, Santa-Maria L et al (2022) The association between maternal ultra-processed food consumption during pregnancy and child neuropsychological development: a population-based birth cohort study. Clin Nutr 41:2275–2283. 10.1016/J.CLNU.2022.08.005 36087519 10.1016/j.clnu.2022.08.005

[42] Trumbo P, Schlicker S, Yates AA, Poos M (2002) Dietary reference intakes for energy, carbohydrate, fiber, fat, fatty acids, cholesterol, protein and amino acids. J Am Diet Assoc 102:1621–1630. 10.1016/S0002-8223(02)90346-9 12449285 10.1016/s0002-8223(02)90346-9

