# Supplementary material for: Exploring the relationship between maternal carbohydrate quality and quantity during pregnancy and early childhood neurodevelopment: a prospective cohort study within the BiSC cohort
Source: Eur J Nutr. 2025 Dec 1;64(8):327. doi: 10.1007/s00394-025-03829-0 (PMC12669324; doi:10.1007/s00394-025-03829-0)
Supplement: Supplementary file 1 — Supplementary file1 (DOCX 122 KB) [file 394_2025_3829_MOESM1_ESM.docx]

**Table S1.** Characteristics of study population according to maternal dietary glycemic index.

|  | T1 (n = 267) | T2 (n = 266) | T3 (n = 267) | p-value |
| --- | --- | --- | --- | --- |
| **Maternal characteristics** (n = 800) | | | | |
| Carbohydrate intake (g/day) | 187 [149, 233] | 193 [158, 239] | 223 [180, 272] | <0.001 |
| Glycemic Index (%) | 39.4 [37, 40.9] | 44.7 [43.7, 46] | 53.3 [50.1, 58] | <0.001 |
| Glycemic Load | 71.2 [58, 91.3] | 86.1 [69.6, 109] | 121 [97.8, 150] | <0.001 |
| Carbohydrate Quality Index | 16 [15, 18] | 14 [13, 16] | 12 [10, 13] | <0.001 |
| Age (years) | 35 [32, 37.6] | 35 [32, 38] | 34 [31, 36] | 0.028 |
| Body Mass Index (kg/m^2^) | 23.6 [21.5, 26.4] | 22.8 [21.1, 25.3] | 22.7 [21.1, 25.4] | 0.077 |
| Total energy intake (kcal/day) | 1826 [1506, 2227] | 1861 [1505, 2227] | 2011 [1660, 2382] | <0.001 |
| Smoking during pregnancy, n (%)  Yes  No | 12 (4.49)  255 (95.51) | 15 (5.64)  251 (94.36) | 15 (5.95)  252 (94.05) | 0.794 |
| Financial status, n (%)  High  Medium or low | 104 (38.95)  163 (61.05) | 96 (36.09)  170 (63.91) | 87 (32.58)  180 (67.42) | 0.307 |
| Education level, n (%)  Secondary school or below  University studies | 64 (23.97)  203 (76.03) | 72 (27.07)  194 (72.93) | 60 (22.47)  207 (77.53) | 0.721 |
| Ethnicity, n (%)  Caucasian  Other | 225 (84.27)  42 (15.73) | 204 (76.69)  62 (23.31) | 210 (78.65)  57 (21.35) | 0.077 |
| Mediterranean Diet Adherence Screener, n (%)  High adherence (≥8)  Low adherence (<8) | 83 (31.09)  184 (68.91) | 116 (43.61)  150 (56.39) | 123 (46.07)  144 (53.93) | <0.001 |
| Pregnancy Physical Activity Questionnaire (MET-h/week) | 162 [118, 219] | 170 [124, 223] | 160 [118, 218] | 0.334 |
| Gestational Diabetes Mellitus, n (%)  Yes  No | 32 (11.98)  235 (88.02) | 16 (6.01)  250 (93.98) | 11 (4.12)  256 (95.88) | 0.001 |
| **Child characteristics** (n = 800) | | | | |
| Sex, n (%)  Male  Female | 147 (55.06)  120 (44.94) | 132 (49.62)  134 (50.38) | 124 (46.44)  143 (53.56) | 0.132 |
| Birth weight (g) | 3310 [3030, 3591] | 3320 [3030, 3600] | 3320 [3038, 3550] | 0.694 |
| Prematurity, n (%)  Yes  No | 8 (3)  259 (97) | 9 (3.38)  257 (96.62) | 11 (4.12)  256 (95.88) | 0.773 |
| Type of lactation at 15 days, n (%)  Breastfeeding  Infant formula  Mixed | 185 (69.29)  20 (7.49)  62 (23.22) | 190 (71.43)  16 (6.01)  60 (22.56) | 196 (73.41)  9 (3.37)  62 (23.22) | 0.344 |
| Delivery type, n (%)  Caesarean  Vaginal | 62 (23.22)  205 (76.78) | 63 (23.68)  203 (76.32) | 68 (25.47)  199 (74.53) | 0.814 |

Numeric data are expressed as median [IQR], while categorical variables are represented as percentatges. P-values are obtained by Kruskall-Wallis test for numeric variables or Chi-Squared test for categorical.

**Table S2.** Characteristics of study population according to maternal dietary glycemic load.

|  | T1 (n = 267) | T2 (n = 266) | T3 (n = 267) | p-value |
| --- | --- | --- | --- | --- |
| **Maternal characteristics** (n = 800) | | | | |
| Carbohydrate intake (g/day) | 147 [129, 166] | 201 [183, 221] | 270 [239, 307] | <0.001 |
| Glycemic Index (%) | 41.5 [38, 44.7] | 44.2 [41.3, 47.9] | 51.1 [45.5, 56.5] | <0.001 |
| Glycemic Load | 62 [53.3, 69.3] | 91.2 [82.5, 97.7] | 132 118, 155] | <0.001 |
| Carbohydrate Quality Index | 14 [12, 16] | 15 [12, 16] | 13 [11.5, 16] | 0.002 |
| Age (years) | 34.6 [31.6, 37.4] | 34.7 [32, 37.6] | 35 [31.8, 37] | 0.968 |
| Body Mass Index (kg/m^2^) | 24 [21.3, 26.6] | 22.7 [21.2, 25.1] | 22.7 21.1, 25.3] | 0.014 |
| Total energy intake (kcal/day) | 1483 [1282, 1705] | 1900 [1668, 2161] | 2360 [2075, 2721] | <0.001 |
| Smoking during pregnancy, n (%)  Yes  No | 12 (4.49)  255 (95.51) | 16 (6.01)  250 (93.98) | 14 (5.24)  253 (94.76) | 0.733 |
| Financial status, n (%)  High  Medium or low | 108 (40.45)  159 (59.55) | 89 (33.46)  177 (66.54) | 90 (33.71)  177 (66.29) | 0.161 |
| Education level, n (%)  Secondary school or below  University studies | 70 (26.22)  197 (73.78) | 59 (22.18)  207 (77.82) | 67 (25.09)  200 (74.91) | 0.802 |
| Ethnicity, n (%)  Caucasian  Other | 219 (82.02)  48 (17.98) | 210 (78.95)  56 (21.05) | 210 (78.65)  57 (21.35) | 0.561 |
| Mediterranean Diet Adherence Screener, n (%)  High adherence (≥8)  Low adherence (<8) | 150 (56.18)  117 (43.82) | 169 (63.53)  97 (36.47) | 159 (59.55)  108 (40.45) | 0.223 |
| Pregnancy Physical Activity Questionnaire (MET-h/week) | 156 [111, 211] | 172 [125, 223] | 163 [120, 223] | 0.071 |
| Gestational Diabetes Mellitus, n (%)  Yes  No | 27 (10.11)  240 (89.89) | 19 (7.14)  247 (92.86) | 13 (4.87)  254 (95.13) | 0.067 |
| **Child characteristics** (n = 800) | | | | |
| Sex, n (%)  Male  Female | 141 (52.81)  126 (47.19) | 125 (46.99)  141 (53.01) | 137 (51.31)  130 (48.69) | 0.378 |
| Birth weight (g) | 3290 [3000, 3560] | 3310 [3030, 3590] | 3345 [3080, 3610] | 0.420 |
| Prematurity, n (%)  Yes  No | 6 (2.25)  261 (97.75) | 10 (3.76)  256 (96.24) | 12 (4.49)  255 (95.51) | 0.354 |
| Type of lactation at 15 days, n (%)  Breastfeeding  Infant formula  Mixed | 194 (72.66)  24 (8.99)  49 (18.35) | 180 (67.67)  13 (4.89)  73 (27.44) | 197 (73.78)  8 (3)  62 (23.22) | 0.006 |
| Delivery type, n (%)  Caesarean  Vaginal | 78 (29.21)  189 (70.79) | 56 (21.05)  210 (78.95) | 59 (22.1)  208 (77.9) | 0.056 |

Numeric data are expressed as median [IQR], while categorical variables are represented as percentatges. P-values are obtained by Kruskall-Wallis test for numeric variables or Chi-Squared test for categorical.

**Table S3.** Characteristics of study population according to maternal dietary carbohydrate quality

index.

|  | T1 (n = 254) | T2 (n = 182) | T3 (n = 364) | p-value |
| --- | --- | --- | --- | --- |
| **Maternal characteristics** (n = 800) | | | | |
| Carbohydrate intake (g/day) | 187 [151, 228] | 193 [157, 244] | 213 [213, 258] | <0.001 |
| Glycemic Index | 52.4 [47.5, 57.8] | 44.5 [41.5, 46.6] | 41.6 [38.5, 44.7] | <0.001 |
| Glycemic Load | 98.6 [73.4, 130] | 83 [66.3, 113] | 90.4 [69.2, 113] | <0.001 |
| Carbohydrate Quality Index | 11 [11, 12] | 14 [14, 14] | 15 [13, 16] | <0.001 |
| Age (years) | 33.6 [31, 36] | 35 [32.1, 38] | 35 [32, 38] | <0.001 |
| Body Mass Index (kg/m^2^) | 22.8 [21, 25.4] | 23.2 [21.5, 25.7] | 23.2 [21.2, 25.8] | 0.550 |
| Total energy intake (kcal/day) | 1757 [1429, 2106] | 1876 [1500, 2290] | 2021 [1640, 2384] | <0.001 |
| Smoking during pregnancy, n (%)  Yes  No | 16 (6.3)  238 (93.7) | 8 (4.4)  174 (95.6) | 18 (4.94)  346 (95.06) | 0.638 |
| Financial status, n (%)  High  Medium or low | 84 (33.07)  170 (66.93) | 69 (37.91)  113 (67.09) | 134 (36.81)  230 (63.19) | 0.513 |
| Education level, n (%)  Secondary school or below  University studies | 70 (27.56)  184 (72.44) | 48 (26.37)  134 (73.63) | 78 (21.43)  286 (78.57) | 0.428 |
| Ethnicity, n (%)  Caucasian  Other | 189 (74.41)  65 (25.59) | 146 (80.22)  36 (19.78) | 304 (83.52)  60 (16.48) | 0.021 |
| Mediterranean Diet Adherence Screener, n (%)  High adherence (≥8)  Low adherence (<8) | 121 (47.64)  133 (52.36) | 120 (65.93)  62 (34.07) | 237 (65.11)  127 (34.87) | <0.001 |
| Pregnancy Physical Activity Questionnaire (MET-h/week) | 150 [114, 208] | 171 [125, 223] | 168 [121, 223] | 0.061 |
| Gestational Diabetes Mellitus, n (%)  Yes  No | 14 (5.51)  240 (94.49) | 11 (6.04)  171 (93.96) | 34 (9.34)  330 (90.66) | 0.148 |
| **Child characteristics** (n = 800) | | | | |
| Sex, n (%)  Male  Female | 119 (48.85)  135 (53.15) | 86 (47.25)  96 (52.75) | 198 (53.4)  166 (45.6) | 0.115 |
| Birth weight (g) | 3272 [2998, 3580] | 3320 [3092, 3585] | 3330 [3040, 3585] | 0.399 |
| Prematurity, n (%)  Yes  No | 9 (3.54)  245 (96.46) | 4 (2.2)  178 (97.8) | 15 (4.12)  349 (95.88) | 0.514 |
| Type of lactation at 15 days, n (%)  Breastfeeding  Infant formula  Mixed | 180 (70.87)  19 (7.48)  55 (21.65) | 127 (69.78)  12 (6.59)  43 (23.63) | 264 (73.53)  14 (3.85)  86 (23.63) | 0.362 |
| Delivery type, n (%)  Caesarean  Vaginal | 68 (26.77)  186 (73.23) | 44 (24.18)  138 (73.63) | 81 (22.25)  283 (77.75) | 0.434 |

Numeric data are expressed as median [IQR], while categorical variables are represented as percentatges. P-values are obtained by Kruskall-Wallis test for numeric variables or Chi-Squared test for categorical.

**Table S4.** Sensitivity associations between dietary carbohydrate intake, GI, GL and CQI during pregnancy and BSID-III infant performance after adjusting for maternal ethnicity.

|  | **Carbohydrate intake** | | | | | |
| --- | --- | --- | --- | --- | --- | --- |
|  | T1 (n=202) | T2 (n=202) | T3 (n=191) | P trend | n = 595 | P-value |
| Cognitive development | Ref. | 1.20 (-1.27, 3.68) | -0.10 (-3.69, 3.50) | 0.913 | 0.01 (-0.02, 0.03) | 0.644 |
| Language development | Ref. | 0.29 (-2.35, 2.94) | -1.53 (-5.38, 2.31) | 0.412 | 1·10^-3^ (-0.03, 0.03) | 0.926 |
| Expressive language | Ref. | 0.05 (-0.37, 0.48) | -0.04 (-0.67, 0.58) | 0.873 | 9·10^-4^ (-4·10^-3^, 0.01) | 0.711 |
| Receptive language | Ref. | -0.03 (-0.55, 0.49) | -0.41 (-1.17, 0.35) | 0.278 | 2·10^-4^ (-0.01, 0.01) | 0.948 |
| Motor development | Ref. | -0.39 (-2.26, 1.48) | -0.56 (-3.29, 2.16) | 0.692 | 0.01 (-0.01, 0.03) | 0.329 |
| Fine motor | Ref. | 0.08 (-0.33, 0.59) | -0.22 (-0.82, 0.38) | 0.451 | -2·10^-4^ (-5·10^-3^, 4·10^-3^) | 0.919 |
| Gross motor | Ref. | -0.25 (-0.62, 0.13) | -0.15 (-0.70, 0.39) | 0.618 | 3·10^-3^ (-2·10^-3^, 0.01) | 0.220 |
|  | **Glycemic Index** | | | | | |
|  | T1 (n=200) | T2 (n=201) | T3 (n=194) | P trend | n = 595 | P-value |
| Cognitive development | Ref. | 0.92 (-1.27, 3.12) | -1.19 (-3.46, 1.08) | 0.231 | -0.02 (-0.14, 0.09) | 0.687 |
| Language development | Ref. | -0.47 (-2.82, 1.88) | -2.06 (-4.49, 0.37) | 0.088 | -0.05 (-0.17, 0.08) | 0.454 |
| Expressive language | Ref. | 0.09 (-0.29, 0.47) | -0.19 (-0.59, 0.20) | 0.277 | -0.01 (-0.03, 0.01) | 0.574 |
| Receptive language | Ref. | -0.25 (-0.71, 0.22) | -0.34 (-0.83, 0.13) | 0.175 | -4·10^-3^ (-0.03, 0.02) | 0.741 |
| Motor development | Ref. | -0.42 (-2.08, 1.24) | -1.39 (-3.11, 0.33) | 0.106 | -0.02 (-0.11, 0.07) | 0.694 |
| Fine motor | Ref. | 0.16 (-0.21, 0.53) | 0.06 (-0.31, 0.44) | 0.810 | 0.01 (-0.01, 0.03) | 0.435 |
| Gross motor | Ref. | -0.27 (-0.60, 0.06) | -0.47 (-0.82, -0.13) | 0.008 | -0.01 (-0.03, 2·10^-3^) | 0.089 |
|  | **Glycemic Load** | | | | | |
|  | T1 (n=201) | T2 (n=201) | T3 (n=193) | P trend | n = 595 | P-value |
| Cognitive development | Ref. | -0.50 (-2.92, 1.93) | -0.81 (-3.90, 2.28) | 0.616 | -2·10^-3^ (-0.04, 0.03) | 0.901 |
| Language development | Ref. | -1.35 (-3.94, 1.24) | -2.18 (-5.49, 1.12) | 0.208 | -0.02 (-0.05, 0.02) | 0.413 |
| Expressive language | Ref. | -0.24 (-0.66, 0.18) | -0.32 (-0.85, 0.21) | 0.266 | -2·10^-3^ (-0.01, 4·10^-3^) | 0.591 |
| Receptive language | Ref. | -0.29 (-0.80, 0.22) | -0.37 (-1.10, 0.28) | 0.299 | -2·10^-3^ (-0.01, 0.01) | 0.680 |
| Motor development | Ref. | -0.42 (-2.26, 1.41) | -0.47 (-2.80, 1.87) | 0.720 | 0.01 (-0.02, 0.03) | 0.623 |
| Fine motor | Ref. | 0.07 (-0.33, 0.48) | -0.06 (-0.58, 0.45) | 0.753 | 2·10^-3^ (-4·10^-3^, 0.01) | 0.546 |
| Gross motor | Ref. | -0.26 (-0.63, 0.11) | -0.24 (-0.71, 0.23) | 0.374 | -6·10^-4^ (-0.01, 5·10^-3^) | 0.810 |
|  | **Carbohydrate Quality Index** | | | | | |
|  | T1 (n=193) | T2 (n=134) | T3 (n=268) | P trend | n = 595 | P-value |
| Cognitive development | Ref. | 0.32 (-2.21, 2.85) | 0.99 (-1.17, 3.15) | 0.403 | 0.20 (-0.14, 0.53) | 0.256 |
| Language development | Ref. | 0.06 (-2.65, 2.77) | 0.82 (-1.49, 3.13) | 0.539 | 0.27 (-0.09, 0.63) | 0.138 |
| Expressive language | Ref. | 0.10 (-0.34, 0.54) | 0.23 (-0.14, 0.60) | 0.255 | 0.04 (-0.02, 0.10) | 0.196 |
| Receptive language | Ref. | -0.14 (-0.67, 0.40) | 0.09 (-0.37, 0.54) | 0.842 | 0.03 (-0.04, 0.10) | 0.372 |
| Motor development | Ref. | 1.81 (-0.10, 3.72) | 0.69 (-0.94, 2.32) | 0.261 | 0.17 (-0.09, 0.42) | 0.196 |
| Fine motor | Ref. | 0.11 (-0.31, 0.53) | -0.13 (-0.49, 0.23) | 0.626 | -0.01 (-0.07, 0.04) | 0.646 |
| Gross motor | Ref. | 0.45 (0.07, 0.84) | 0.36 (0.03, 0.69) | 0.017 | 0.07 (0.02, 0.12) | 0.008 |

Results are expressed as β coefficients and 95% confidence interval estimated using linear model adjused for child sex and age at behavioral assesment and materanl age at enrollment, financial status, education, pregnancy body mass index, physical activity, pregnancy smoking status, adherence to Mediterranean diet (MEDAS), maternal energy intake, gestational diabetes mellitus, child weight at birth, prematurity, type of lactation, type of delivery and maternal ethnicity. Abbreviations: GI, glycemic index; GL, glycemic load; CQI, carbohydrate quality index; BSID-III, Bayley Scales of Infant and Toddler Development.

**Table S5.** Sensitivity associations between dietary carbohydrate intake, GI, GL and CQI during pregnancy and BSID-III infant performance after adjusting for child type of feeding at 18.

|  | **Carbohydrate intake** | | | | | |
| --- | --- | --- | --- | --- | --- | --- |
|  | T1 (n=167) | T2 (n=173) | T3 (n=157) | P trend | n =497 | P-value |
| Cognitive development | Ref. | 1.21 (-1.61, 4.03) | 0.16 (-3.94, 4.25) | 0.992 | 5·10^-3^ (-0.03, 0.04) | 0.783 |
| Language development | Ref. | -0.56 (-3.55, 2.42) | -2.67 (-7.00, 1.67) | 0.215 | -0.02 (-0.06, 0.01) | 0.242 |
| Expressive language | Ref. | -0.09 (-0.56, 0.38) | -0.18 (-0.87, 0.50) | 0.600 | -1·10^-3^ (-0.01, 4·10^-3^) | 0.596 |
| Receptive language | Ref. | -0.19 (-0.78, 0.40) | -0.64 (-1.50, 0.22) | 0.135 | -5·10^-3^ (-0.01, 2·10^-3^) | 0.188 |
| Motor development | Ref. | 0.05 (-2.19, 2.08) | 0.06 (-3.16, 3.04) | 0.969 | 4·10^-3^ (-0.02, 0.03) | 0.737 |
| Fine motor | Ref. | 0.04 (-0.44, 0.51) | -0.24 (-0.92, 0.45) | 0.468 | -3·10^-3^ (-0.01, 3·10^-3^) | 0.354 |
| Gross motor | Ref. | -0.09 (-0.51, 0.33) | 0.01 (-0.60, 0.62) | 0.948 | 3·10^-3^ (-2·10^-3^, 0.01) | 0.264 |
|  | **Glycemic Index** | | | | | |
|  | T1 (n=167) | T2 (n=171) | T3 (n=159) | P trend | n =497 | P-value |
| Cognitive development | Ref. | 0.06 (-2.42, 2.54) | -1.33 (-3.92, 1.25) | 0.280 | -0.02 (-0.15, 0.11) | 0.776 |
| Language development | Ref. | -1.22 (-3.84, 1.40) | -2.38 (-5.11, -0.36) | 0.092 | -0.05 (-0.19, 0.09) | 0.477 |
| Expressive language | Ref. | -0.06 (-0.48, 0.35) | -0.25 (-0.68, 0.18) | 0.242 | -4·10^-3^ (-0.03, 0.02) | 0.700 |
| Receptive language | Ref. | -0.35 (-0.87, 0.17) | -0.36 (-0.90, 0.18) | 0.232 | -5·10^-3^ (-0.03, 0.02) | 0.722 |
| Motor development | Ref. | -0.86 (-2.73, 1.02) | -1.62 (-3.58, 0.33) | 0.107 | -0.03 (-0.13, 0.07) | 0.578 |
| Fine motor | Ref. | 0.04 (-0.37, 0.46) | 0.01 (-0.42, 0.44) | 0.982 | 0.01 (-0.02, 0.03) | 0.623 |
| Gross motor | Ref. | -0.30 (-0.66, 0.07) | -0.51 (-0.89, -0.12) | 0.012 | -0.02 (-0.04, 2·10^-3^) | 0.084 |
|  | **Glycemic Load** | | | | | |
|  | T1 (n=169) | T2 (n=173) | T3 (n=155) | P trend | n =497 | P-value |
| Cognitive development | Ref. | -1.05 (-3.81, 1.70) | -1.39 (-4.94, 2.16) | 0.468 | -0.01 (-0.05, 0.04) | 0.793 |
| Language development | Ref. | -2.50 (-5.40, 0.41) | -4.13 (-7.88, -0.38) | 0.036 | -0.03 (-0.08, 0.01) | 0.142 |
| Expressive language | Ref. | -0.44 (-0.90, 0.02) | -0.62 (-1.21, -0.03) | 0.052 | -2·10^-3^ (-0.01, 4·10^-3^) | 0.476 |
| Receptive language | Ref. | -0.50 (-1.07, 0.08) | -0.72 (-1.46, 0.02) | 0.069 | -0.01 (-0.01, 3·10^-3^) | 0.193 |
| Motor development | Ref. | -0.43 (-2.51, 1.66) | -0.23 (-2.92, 2.45) | 0.897 | -4·10^-3^ (-0.03, 0.03) | 0.810 |
| Fine motor | Ref. | -0.07 (-0.54, 0.39) | -0.12(-0.72, 0.47) | 0.695 | -7·10^-4^ (-0.01, 0.01) | 0.836 |
| Gross motor | Ref. | -0.11 (-0.52, 0.30) | -0.13 (-0.66, 0.40) | 0.656 | -2·10^-3^ (-0.01, 4·10^-3^) | 0.554 |
|  | **Carbohydrate Quality Index** | | | | | |
|  | T1 (n=156) | T2 (n=117) | T3 (n=224) | P trend | n =497 | P-value |
| Cognitive development | Ref. | -0.11 (-2.98, 2.77) | 1.19 (-1.29, 3.68) | 0.420 | 0.28 (-0.11, 0.66) | 0.159 |
| Language development | Ref. | 0.10 (-2.95, 3.15) | 0.79 (-1.85, 3.42) | 0.600 | 0.32 (-0.09, 0.72) | 0.127 |
| Expressive language | Ref. | 0.07 (-0.41, 0.55) | 0.26 (-0.15, 0.68) | 0.249 | 0.05 (-0.02, 0.11) | 0.159 |
| Receptive language | Ref. | -0.11 (-0.72, 0.49) | 0.03 (-0.49, 0.56) | 0.972 | 0.03 (-0.05, 0.12) | 0.397 |
| Motor development | Ref. | 1.77 (-0.40, 3.94) | 0.59 (-1.29, 2.46) | 0.384 | 0.19 (-0.10, 0.48) | 0.194 |
| Fine motor | Ref. | 0.12 (-0.36, 0.61) | -0.11 (-0.53, 0.31) | 0.727 | -3·10^-3^ (-0.07, 0.06) | 0.936 |
| Gross motor | Ref. | 0.42 (-0.01, 0.85) | 0.31 (-0.06, 0.68) | 0.069 | 0.07 (0.01, 0.12) | 0.023 |

Results are expressed as β coefficients and 95% confidence interval estimated using linear model adjused for child sex and age at behavioral assesment and materanl age at enrollment, financial status, education, pregnancy body mass index, physical activity, pregnancy smoking status, adherence to Mediterranean diet (MEDAS), maternal energy intake, gestational diabetes mellitus, child weight at birth, prematurity, type of lactation at 18 months of age and type of delivery. Abbreviations: GI, glycemic index; GL, glycemic load; CQI, carbohydrate quality index; BSID-III, Bayley Scales of Infant and Toddler Development.

**Table S6.** Sensitivity associations between dietary carbohydrate, GI, GL and CQI intake during pregnancy and DP-3 infant performance after maternal ethnicity adjustment.

|  | **Carbohydrate intake** | | | | | |
| --- | --- | --- | --- | --- | --- | --- |
|  | T1 (n= 100) | T2 (n=119) | T3 (n=107) | P trend | n = 326 | P-value |
| Global Development Index | Ref. | 0.41 (-2.60, 3.43) | 1.06 (-3.14, 5.26) | 0.624 | -0.02 (-0.06, 0.01) | 0.224 |
| Adaptive Behavior | Ref. | -0.10 (-2.55, 2.35) | 1.45 (-1.97, 4.87) | 0.359 | -0.01 (-0.04, 0.02) | 0.589 |
| Social–Emotional | Ref. | 0.66 (-2.15, 3.47) | 0.54 (-3.38, 4.46) | 0.833 | -0.02 (-0.05, 0.02) | 0.311 |
| Cognitive | Ref. | 0.84 (-1.99, 3.67) | -0.05 (-3.99, 3.89) | 0.895 | -0.02 (-0.05, 0.01) | 0.183 |
| Communication | Ref. | 1.51 (-1.59, 4.61) | 0.87 (-3.45, 5.20) | 0.796 | -0.02 (-0.05, 0.02) | 0.303 |
| Motor development | Ref. | -1.74 (-4.41, 0.93) | 0.73 (-2.99, 4.44) | 0.520 | -0.02 (-0.05, 0.01) | 0.298 |
|  | **Glycemic Index** | | | | | |
|  | T1 (n=116) | T2 (n=104) | T3 (n=106) | P trend | n = 326 | P-value |
| Global Development Index | Ref. | 0.97 (-1.66, 3.41) | -1.49 (-4.15, 1.17) | 0.246 | -0.08 (-0.22, 0.07) | 0.293 |
| Adaptive Behavior | Ref. | 0.73 (-1.34, 2.80) | -1.39 (-3.55, 0.78) | 0.188 | -0.09 (-0.21, 0.02) | 0.121 |
| Social–Emotional | Ref. | 0.92 (-1.45, 3.30) | -0.39 (-2.88, 2.09) | 0.708 | -0.04 (-0.18, 0.09) | 0.525 |
| Cognitive | Ref. | 0.80 (-1.59, 3.19) | -0.28 (-2.78, 2.23) | 0.786 | 0.01 (-0.12, 0.14) | 0.880 |
| Communication | Ref. | 1.20 (-1.42, 3.83) | -0.08 (-3.83, 2.66) | 0.894 | -0.01 (-0.16, 0.13) | 0.856 |
| Motor development | Ref. | -0.25 (-2.49, 1.99) | -3.33 (-5.68, -0.99) | 0.006 | -0.14 (-0.26, -0.01) | 0.033 |
|  | **Glycemic Load** | | | | | |
|  | T1 (n=108) | T2 (n=109) | T3 (n=109) | P trend | n = 326 | P-value |
| Global Development Index | Ref. | 1.76 (-1.11, 4.62) | -1.38 (-5.00, 2.24) | 0.333 | -0.04 (-0.08, 0.01) | 0.094 |
| Adaptive Behavior | Ref. | 1.19 (-1.14, 3.52) | -1.75 (-4.70, 1.20) | 0.169 | -0.03 (-0.06, 0.01) | 0.122 |
| Social–Emotional | Ref. | 2.31 (-0.35, 4.97) | -1.30 (-4.66, 2.06) | 0.294 | -0.03 (-0.07, 0.01) | 0.146 |
| Cognitive | Ref. | 2.68 (-5·10^-4^, 5.36) | -0.32 (-3.71, 3.07) | 0.627 | -0.02 (-0.06, 0.02) | 0.385 |
| Communication | Ref. | 0.72 (-2.25, 3.69) | -0.27 (-4.03, 3.48) | 0.824 | -0.02 (-0.06, 0.02) | 0.358 |
| Motor development | Ref. | -1.04 (-3.61, 1.53) | -1.35 (-4.60, 1.89) | 0.455 | -0.04 (-0.07, -3·10^-4^) | 0.049 |
|  | **Carbohydrate Quality Index** | | | | | |
|  | T1 (n=99) | T2 (n=75) | T3 (n=152) | P trend | n = 326 | P-value |
| Global Development Index | Ref. | 0.76 (-2.23, 3.75) | 1.20 (-1.40, 3.80) | 0.386 | 0.25 (-0.16, 0.66) | 0.229 |
| Adaptive Behavior | Ref. | 0.89 (-1.55, 3.32) | 1.56 (-0.37, 3.84) | 0.172 | 0.23 (-0.10, 0.56) | 0.181 |
| Social–Emotional | Ref. | -1.54 (-4.33, 1.24) | -0.81 (-3.24, 1.61) | 0.433 | 0.05 (-0.33, 0.43) | 0.812 |
| Cognitive | Ref. | 0.23 (-3.04, 2.58) | 0.30 (-2.15, 2.74) | 0.866 | 0.09 (-0.29, 0.47) | 0.640 |
| Communication | Ref. | 1.42 (-1.66, 4.51) | 0.64 (-2.04, 3.32) | 0.557 | 0.12 (-0.30, 0.54) | 0.564 |
| Motor development | Ref. | 2.25 (-0.40, 4.90) | 2.50 (0.20, 4.80) | 0.032 | 0.41 (0.05, 0.77) | 0.026 |

Results are expressed as β coefficients and 95% confidence interval estimated using linear mixed model adjused for child sex and age at behavioral assesment and materanl age at enrollment, financial status, education, pregnancy body mass index, physical activity, pregnancy smoking status, adherence to Mediterranean diet (MEDAS), maternal energy intake, gestational diabetes mellitus, child weight at birth, prematurity, type of lactation, type of delivery and maternal ethnicity. Abbreviations: GI, glycemic index; GL, glycemic load; CQI, carbohydrate quality index; DP-3, Developmental Profile 3.

**Table S7.** Sensitivity associations between dietary carbohydrate, GI, GL and CQI intake during pregnancy and DP-3 infant performance after adjusting for type of feeding at 18 months.

|  | **Carbohydrate intake** | | | | | |
| --- | --- | --- | --- | --- | --- | --- |
|  | T1 (n=93) | T2 (n=112) | T3 (n=94) | P trend | n = 299 | P-value |
| Global Development Index | Ref. | 0.58 (-2.49, 3.64) | 0.76 (-3.61, 5.13) | 0.757 | -0.03 (-0.06, 0.01) | 0.145 |
| Adaptive Behavior | Ref. | 0.23 (-2.33, 2.79) | 1.41 (-2.25, 5.06) | 0.436 | -0.01 (-0.04, 0.02) | 0.476 |
| Social–Emotional | Ref. | 0.77 (-2.07, 3.62) | 0.27 (-3.79, 4.33) | 0.950 | -0.02 (-0.05, 0.01) | 0.280 |
| Cognitive | Ref. | 1.28 (-1.58, 4.13) | -0.21 (-4.28, 3.86) | 0.815 | -0.02 (-0.06, 0.01) | 0.151 |
| Communication | Ref. | 1.16 (-1.94, 4.27) | 0.13 (-4.29, 4.55) | 0.964 | -0.03 (-0.06, 0.01) | 0.159 |
| Motor development | Ref. | -1.62 (-4.43, 1.20) | 0.86 (-3.15, 4.88) | 0.541 | -0.02 (-0.05, 0.01) | 0.227 |
|  | **Glycemic Index** | | | | | |
|  | T1 (n=106) | T2 (n=96) | T3 (n=97) | P trend | n = 299 | P-value |
| Global Development Index | Ref. | 0.70 (-1.93, 3.33) | -2.29 (-5.05, 0.46) | 0.095 | -0.10 (-0.25, 0.04) | 0.174 |
| Adaptive Behavior | Ref. | 0.55 (-1.65, 2.75) | -1.91 (-4.21, 0.40) | 0.098 | -0.11 (-0.23, 0.01) | 0.071 |
| Social–Emotional | Ref. | 0.60 (-1.84, 3.05) | -1.68 (-4.25, 0.88) | 0.185 | -0.09 (-0.22, 0.05) | 0.195 |
| Cognitive | Ref. | 0.59 (-1.88, 3.06) | -0.55 (-3.14, 2.04) | 0.652 | 4·10^-3^ (-0.13, 0.14) | 0.949 |
| Communication | Ref. | 1.36 (-1.32, 4.03) | -0.66 (-3.46, 2.14) | 0.592 | -0.03 (-0.17, 0.12) | 0.731 |
| Motor development | Ref. | -0.26 (-2.67, 2.15) | -3.50 (-6.03, -0.98) | 0.007 | -0.14 (-0.27, -0.01) | 0.041 |
|  | **Glycemic Load** | | | | | |
|  | T1 (n=100) | T2 (n=103) | T3 (n=96) | P trend | n = 299 | P-value |
| Global Development Index | Ref. | 1.34 (-1.58, 4.26) | -2.24 (-6.00, 1.52) | 0.183 | -0.04 (-0.09, -6·10^-4^) | 0.048 |
| Adaptive Behavior | Ref. | 1.21 (-1.23, 3.65) | -2.08 (-5.23, 1.06) | 0.140 | -0.03 (-0.07, 3·10^-3^) | 0.070 |
| Social–Emotional | Ref. | 2.07 (-0.62, 4.77) | -2.19 (-5.66, 1.28) | 0.141 | -0.04 (-0.08, -2·10^-5^) | 0.051 |
| Cognitive | Ref. | 2.48 (-0.24, 5.20) | -0.86 (-4.36, 2.65) | 0.467 | -0.02 (-0.06, 0.02) | 0.339 |
| Communication | Ref. | -0.22 (-3.20, 2.76) | -1.46 (-5.31, 2.38) | 0.445 | -0.03 (-0.07, 0.02) | 0.250 |
| Motor development | Ref. | -1.10 (-3.82, 1.62) | -1.44 (-4.94, 2.06) | 0.459 | -0.04 (-0.08, -4·10^-4^) | 0.048 |
|  | **Carbohydrate Quality Index** | | | | | |
|  | T1 (n=92) | T2 (n=68) | T3 (n=139) | P trend | n = 299 | P-value |
| Global Development Index | Ref. | 1.38 (-1.72, 4.49) | 1.61 (-1.05, 4.27) | 0.238 | 0.27 (-0.15, 0.69) | 0.206 |
| Adaptive Behavior | Ref. | 1.11 (-1.48, 3.70) | 1.78 (-0.45, 4.00) | 0.134 | 0.22 (-0.13, 0.57) | 0.216 |
| Social–Emotional | Ref. | 0.42 (-3.31, 2.48) | -0.28 (-2.76, 2.20) | 0.806 | 0.12 (-0.27, 0.51) | 0.540 |
| Cognitive | Ref. | 0.07 (-2.83, 2.98) | 0.74 (-1.75, 3.23) | 0.614 | 0.10 (-0.29, 0.49) | 0.619 |
| Communication | Ref. | 1.70 (-1.44, 4.85) | 1.10 (-1.60, 3.80) | 0.372 | 0.15 (-0.27, 0.58) | 0.476 |
| Motor development | Ref. | 2.48 (-0.38, 5.33) | 2.30 (-0.15, 4.75) | 0.057 | 0.37 (-0.02, 0.75) | 0.063 |

Results are expressed as β coefficients and 95% confidence interval estimated using linear mixed model adjused for child sex and age at behavioral assesment and materanl age at enrollment, financial status, education, pregnancy body mass index, physical activity, pregnancy smoking status, adherence to Mediterranean diet (MEDAS), maternal energy intake, gestational diabetes mellitus, child weight at birth, prematurity, type of lactation at 18 months old and type of delivery. Abbreviations: GI, glycemic index; GL, glycemic load; CQI, carbohydrate quality index; DP-3, Developmental Profile 3.

**Table S8.** Sensitivity associations between dietary GI, GL and CQI intake during pregnancy and cognitive performance after adjusting for maternal fat intake.

| BSID-III | | | | | | | | | |  |
| --- | --- | --- | --- | --- | --- | --- | --- | --- | --- | --- |
|  | | Tertiles of dietary GI | | |  | GI | |  | |  |
|  |  | T1 | T2 | T3 | P trend |  | | P value | |  |
| Language development | | Ref. | -0.78 (-3.24, 1.68) | -2.34 (-5.01, 0.32) | 0.08 | -0.05 (-0.19, 0.09) | | 0.48 | |  |
| Receptive language | | Ref. | -0.30 8-0.79, 0.19) | -0.36 (-0.89, 0.17) | 0.20 | -0.002 (-0.03, 03) | | 0.07 | |  |
| Motor development | | Ref. | -0.89 (-2.59, 0.81) | -2.17 (-4.01, -0.32) | 0.02 | -0.05 (-0.15, 0.04) | | 0.28 | |  |
| Gross motor | | Ref. | -0.35 (-0.69, -0.01) | -0.62 (-0.98, -0.25) | 0.001 | -0.02 (-0.04, -0.003) | | 0.02 | |  |
|  | | Tertiles of dietary GL | | |  | GL | |  | |  |
|  |  | T1 | T2 | T3 | P trend |  | | P value | |  |
| Language development | | Ref. | -2.04 (-4.87, 0.79) | -3.50 (-7.59, 0.59) | 0.10 | -0.03 (-0.08, 0.02) | | 0.28 | |  |
| Receptive language | | Ref. | -0.38 (-0.94, 0.18) | -0.49 (-1.30, 0.33) | 0.27 | -0.001 (-0.01, 0.01) | | 0.88 | |  |
|  | | Tertiles of dietary CQI | | |  | CQI | |  | |  |
|  |  | T1 | T2 | T3 | P trend |  | | P value | |  |
| Gross motor | | Ref. | 0.49 (0.10, 0.87) | 0.42 (0.09, 0.75) | 0.01 | 0.08 (0.03, 0.13) | | 0.003 | |  |
| DP-3 | | | | | | | | | | |
|  | Tertiles of dietary GI | | | |  | | GI | |  | |
|  | T1 | | T2 | T3 | P trend | |  | | P value | |
| Motor development | Ref. | | -0.10 (-2.40, 2.19) | -3.02 (-5.57, -0.47) | 0.02 | | -0.12 (-0.26, 0.02) | | 0.09 | |
|  | Tertiles of dietary CQI | | | |  | | CQI | |  | |
|  | T1 | | T2 | T3 | P trend | |  | | P value | |
| Motor development | Ref. | | 2.09 (-0.64, 4.82) | 2.04 (-0.31, 4.38) | 0.08 | | 0.35 (-0.02, 0.71) | | 0.06 | |

Results are expressed as β coefficients and 95% confidence interval estimated using linear model. Adjusted for child sex and age at behavioral assessment and mother age at enrollment, financial status, education, pregnancy body mass index, physical activity, pregnancy smoking status, adherence to Mediterranean diet (MEDAS), maternal energy intake and maternal gestational diabetes, child weight at birth, prematurity, type of lactation, type of delivery and maternal saturated, monounsaturated and polyunsaturated fat intake. Abbreviations: GI, glycemic index; GL, glycemic load; CQI, carbohydrate quality index; BSID-III, Bayley Scales of Infant Development third edition; DP-3, Developmental Profile 3.
