# Supplementary figures and images for: Exploring the relationship between maternal carbohydrate quality and quantity during pregnancy and early childhood neurodevelopment: a prospective cohort study within the BiSC cohort
Source: Eur J Nutr. 2025 Dec 1;64(8):327. doi: 10.1007/s00394-025-03829-0 (PMC12669324; doi:10.1007/s00394-025-03829-0)

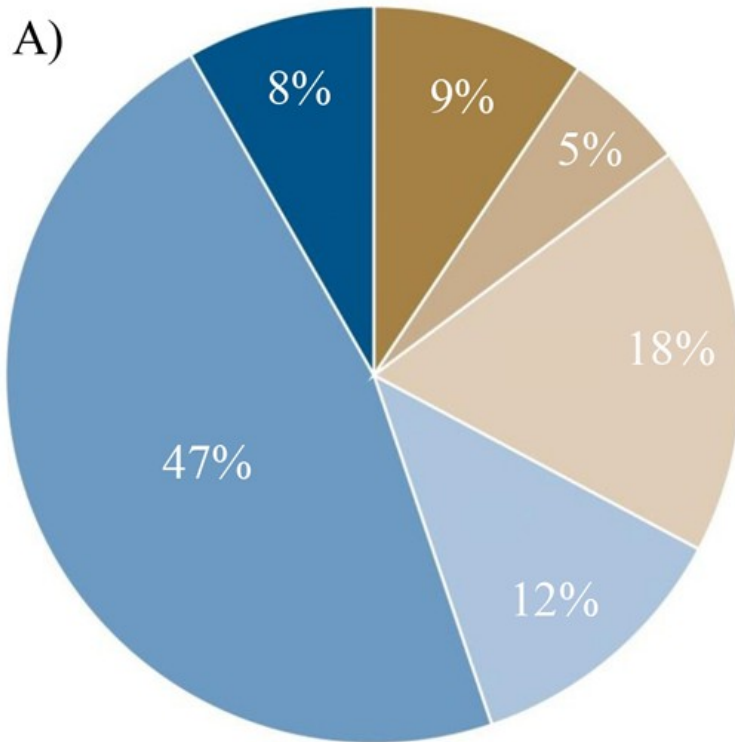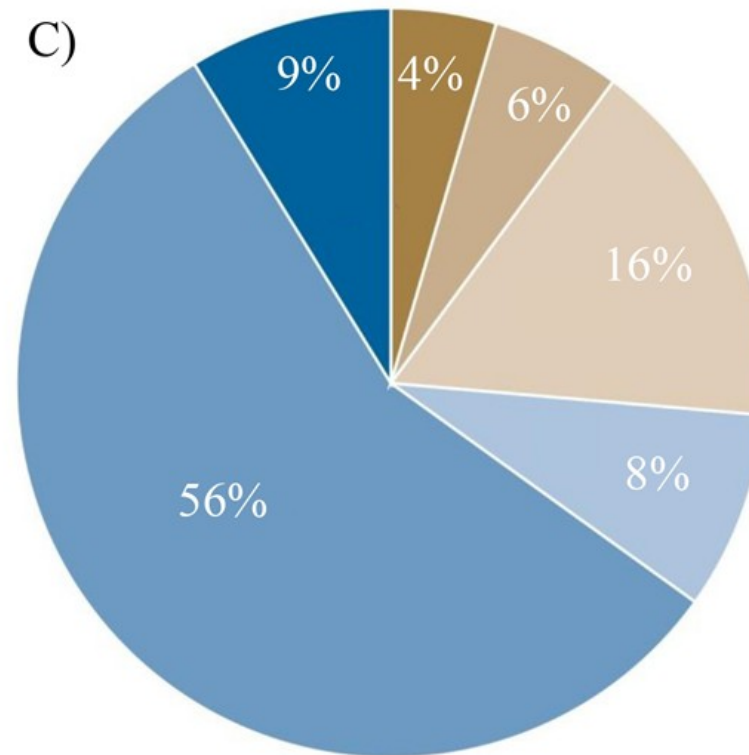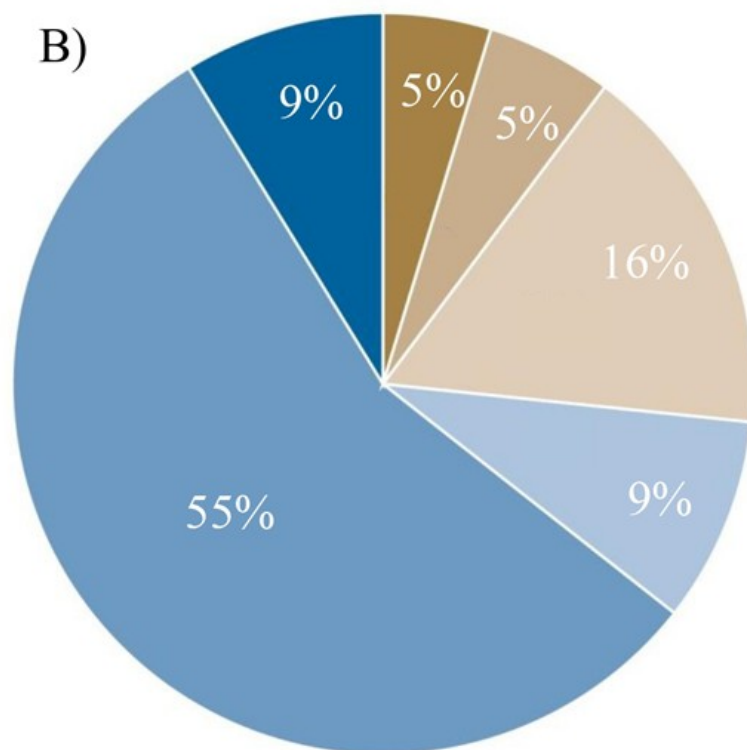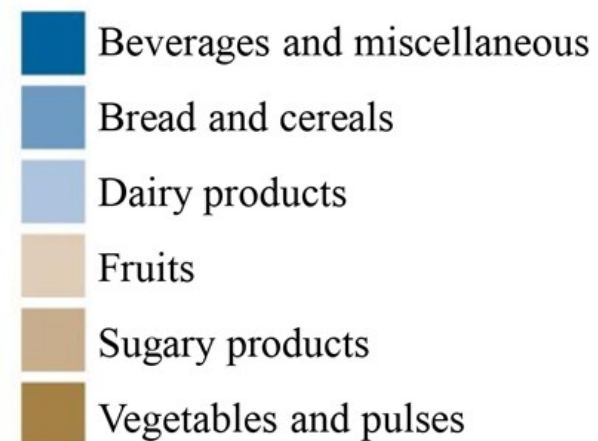

Supplement: Supplementary file 2 — Figure S1. Food groups contribution to maternal A) total carbohydrate intake, B) dietary glycemic index and C) dietary glycemic load during pregnancy. Supplementary file2 (PDF 101 KB) [file 394_2025_3829_MOESM2_ESM.pdf]
